# Modelling the impact of wastewater flows and management practices on antimicrobial resistance in dairy farms

**DOI:** 10.1038/s44259-024-00029-4

**Published:** 2024-05-14

**Authors:** Henry Todman, Richard Helliwell, Liz King, Adam Blanchard, Charlotte J. Gray-Hammerton, Steven P. Hooton, Michelle Baker, Jean Margerison, Paul Wilson, Christine E. R. Dodd, Carol Morris, Sujatha Raman, Chris Hudson, Jan-Ulrich Kreft, Jon L. Hobman, Theodore Kypraios, Dov J. Stekel

**Affiliations:** 1 School of Biosciences, University of Nottingham, Sutton Bonington Campus, College Road, Loughborough, Leicestershire LE12 5RD UK; 2 School of Geography, University of Nottingham, University Park Campus, Nottingham, NG7 2RD UK; 3 School of Sociology and Social Policy, University of Nottingham, University Park Campus, Nottingham, NG7 2RD UK; 4 Ruralis, University Centre Dragvoll, N—7491 Trondheim, Norway; 5 School of Veterinary Medicine and Science, University of Nottingham, Sutton Bonington Campus, Loughborough, Leicestershire LE12 5RD UK; 6 Ineos Oxford Institute for Antimicrobial Research, Sir William Dunn School of Pathology, University of Oxford, South Parks Road, Oxford, OX1 3RE UK; 7 Department of Genetics and Genome Biology, University of Leicester, University Road, Leicester, LE1 7RH UK; 8 Australian National Centre for Public Awareness of Science, Australian National University, Canberra, Australia; 9 Institute of Microbiology and Infection & School of Biosciences, University of Birmingham, Edgbaston, Birmingham, B15 2TT UK; 10 School of Mathematical Sciences, University of Nottingham, University Park Campus, Nottingham, NG7 2RD UK; 11 Department of Mathematics and Applied Mathematics, University of Johannesburg, Auckland Park Kingsway Campus, Rossmore, Johannesburg South Africa

**Keywords:** Antimicrobial resistance, Computational models

## Abstract

Dairy slurry is a major source of environmental contamination with antimicrobial resistant genes and bacteria. We developed mathematical models and conducted on-farm research to explore the impact of wastewater flows and management practices on antimicrobial resistance (AMR) in slurry. Temporal fluctuations in cephalosporin-resistant *Escherichia coli* were observed and attributed to farm activities, specifically the disposal of spent copper and zinc footbath into the slurry system. Our model revealed that resistance should be more frequently observed with relevant determinants encoded chromosomally rather than on plasmids, which was supported by reanalysis of sequenced genomes from the farm. Additionally, lower resistance levels were predicted in conditions with lower growth and higher death rates. The use of muck heap effluent for washing dirty channels did not explain the fluctuations in cephalosporin resistance. These results highlight farm-specific opportunities to reduce AMR pollution, beyond antibiotic use reduction, including careful disposal or recycling of waste antimicrobial metals.

## Introduction

Antimicrobial resistance (AMR) is one of the most important global public health problems. It is estimated that 1.27 million deaths were attributed to AMR bacteria globally in 2019^1^, and, unless suitable countermeasures are taken, that number is predicted to rise to 10 million by 2050^2^. AMR is driven by antibiotic use; the majority (73%) of antibiotic (Ab) sales are for use for food-producing livestock^3^. The use of Abs in agriculture can result in drug-resistant strains infecting human populations through the food chain^4,5^, or may lead to the transfer of antibiotic resistance genes (ARGs) from livestock-associated bacteria to human-acquired infections^6–8^. The importance of mitigating the risks of AMR in the agricultural sector has been recognised by many countries, including the UK, the European Union and the UN^2,9^, with reductions and restrictions being imposed on Ab use in agriculture, particularly on human critical antibiotics. However, despite a 55% reduction in Ab use in the UK agriculture sector since 2014^10^, use remains high, representing 36% of the total UK Ab use^11^, with consequent risk of spread of ARGs and AMR.

In addition to antibiotics, other antimicrobials such as metals (copper and zinc) and other chemicals (e.g., formalin, disinfectants) are widely used across farms globally, particularly in footbaths to prevent lameness in livestock - a prevalent concern in dairy and sheep farming^12^. Metals and other antimicrobial agents (such as formalin and glutaraldehyde) are known to have a co-selective effect on antibiotic resistance, allowing for the persistence of antimicrobial resistance bacteria (ARB) in the absence of antibiotic selective pressures^13–19^.

Cattle account for approximately 50% of global livestock (by Livestock Standard Units)^20^ including approximately 265 million dairy cows (www.faostat.org). These are estimated to produce 3 billion tonnes of manure per year. This study is based in the UK, whose agriculture sector produces approximately 83 million tonnes of livestock manure each year, with a significant amount of this due to dairy cattle farming (28 million tonnes) where 63% of the dairy waste produced is undiluted liquid slurry^21^. Liquid slurry is often stored in slurry tanks or lagoons for several months, principally to avoid spreading them on land in autumn and winter due to restrictions to avoid agricultural nitrate pollution. Dairy slurry has been shown to contain bacteria resistant to many antibiotics, including penicillins, cephalosporins, aminoglycosides, quinolones, sulphonamides, phenicols, tetracyclines, and nitrofurans^22^, which have been associated with current or previous farm antibiotic use^23^. Importantly, dairy slurry can include Extended Spectrum Cephalosporin Resistant *E. coli* (ESCR-EC)^24^, for example AmpC overexpression strains, or Extended Spectrum Beta-Lactamase producing *E. coli* (ESBL-EC), the WHO’s recommended indicator for global surveillance of AMR^25^. The spreading of slurry/manure onto field soil as fertiliser may then release ARGs and ARBs into the surrounding environment, consequently allowing for potential transmission to human pathogens, or to humans via the food chain^26^. Studies of fields that have been spread with dairy slurry have demonstrated increased levels of antibiotics and ARGs present^27–29^. Similar studies have shown that crops fertilised with manure can accumulate ARGs associated with the slurry^27,30–32^.

Thus, there is a clear need to reduce AMR contamination from agricultural waste. However, further reduction in usage of Ab in commercial livestock farming will be extremely challenging for countries such as the UK, that have already made major reductions, due to the need for targeted antibiotic treatment use, whether viewed from an animal welfare or from a farm business perspective. Therefore, it is appropriate to consider whether changes in farm management, infrastructure, or practice, can reduce selection for resistance^23^. Such changes are often difficult to evaluate empirically, because they would need expensive changes to infrastructure, or changes in management practice, with consequent welfare or business risks. Mathematical modelling is a powerful tool in such studies, because alternative strategies can be readily evaluated through simulations, and parameters or processes to which adverse outcomes (i.e., proliferation of ARBs) are especially sensitive can be identified, which serve as potential points of control^23^.

Most mathematical models studying the impact of AMR in dairy farms (or other livestock farm environments) consider a single area of a farm^23,33,34^, treat the entire farm as a single compartment^35^, or are interested in within-host dynamics of the livestock^36^. While such approaches are undoubtedly useful, to the best of our knowledge, there are no modelling studies that investigate the effects of farm layout, the farm practices associated across different areas of the farm, and the impact these may have upon the emergence and/or spread of AMR across the farm.

In this study, we specifically aim to understand how fluctuations in important ARBs could arise as a result of farm infrastructure and practice. This is motivated by previous empirical work, in which we observed the sporadic appearance of ESCR-EC in the slurry tank^23^. At the core of this study is the development and analysis of a multi-scale whole-farm mathematical model for AMR that describes the flow of wastewater around a dairy farm, and the spread of resistance within and between farm compartments. In order to develop the model, we have carried out anthropological research on farm management practice on a typical high performance dairy farm that has uncovered important details of farm operations, which are then incorporated into the model. Moreover, we report additional microbiological measurements on *E. coli* counts in different farm locations, in order to deepen our understanding of the farm microbiology. We used the model to explain ARB outcomes and fluctuations, testing hypotheses derived from the anthropological and microbiological data, by using sensitivity analyses and counterfactual simulations. We also test whether resistance levels will depend upon plasmid or chromosomal carriage of genes conferring cephalosporin resistance. In this way, we show how an interdisciplinary approach, combining mathematical modelling, anthropology and microbiology, can show how farm-scale wastewater flows and management practices can have a material impact on AMR at both population and molecular genetic levels.

## Results

### Farm practices led to high variability in bacterial load across the farm

**Fig. 1:**
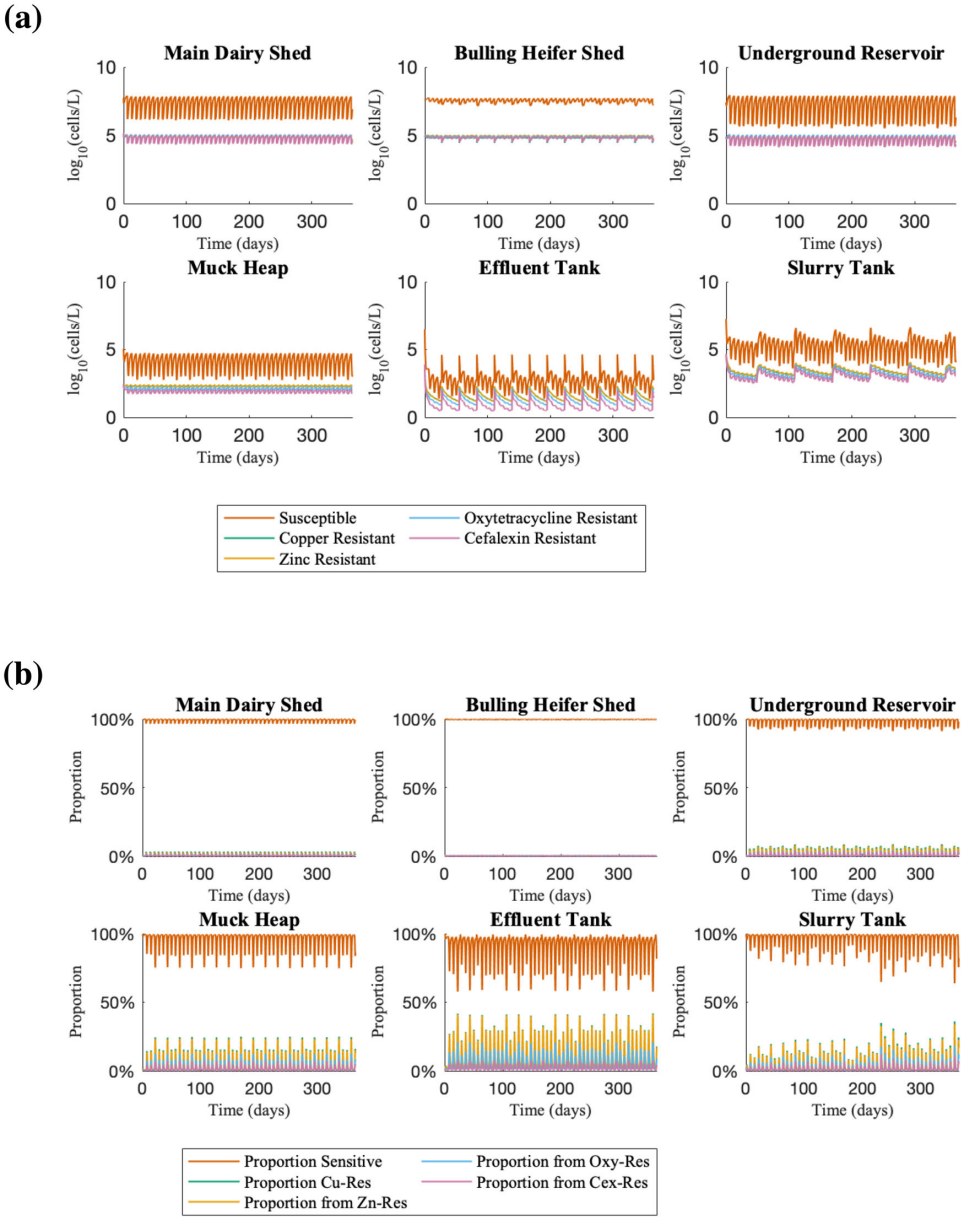
Time course simulations of farm flow model where resistance is plasmid-encoded. **a** Time course simulation showing the concentration of bacterial populations across different areas of the farm over 365 days, using parameter values gathered from farm data (Supplementary Tables 1–6). Resistance genes to oxytetracycline, cefalexin, copper and zinc are assumed to be encoded on plasmids and may be transferred horizontally between populations. Copper resistance is very similar to zinc resistance and so is not visible. **b** The same simulation, plotting the proportions of each sub-population (sensitive, Cu-, Zn-, Oxy- and Cex-resistant bacteria).

Time course simulation of the farm flow model (Fig. 1a) using the default parameter set (Supplementary Tables 1–6) showed that all resistances (zinc, copper, oxytetracycline and cefalexin) are consistently present, commensurate with the slurry tank only simulations^23^, but also showed high variability in the bacterial populations corresponding to different farm practices. There are three timescales affecting the dynamics. The first timescale are fluctuations with a frequency of 7 days in all bacterial populations across the dairy shed, bulling heifer shed, underground reservoir and slurry tank, associated with the weekly emptying of the metal containing footbaths into the scraper channels, as this causes substantial increases in the metal concentration within the slurry (Cu and Zn increases by up to 115-fold and 38-fold respectively in the main dairy shed). This results in increased bacterial death due to the antimicrobial effects of copper and zinc, as the total bacterial concentration in the main dairy shed falls by approximately 98% each time the footbaths are emptied. Copper and zinc resistances are also correlated because of the correlated selection pressure from the footbaths.

The second timescale is associated with the use of additional metal containing footbaths every 21 days (more visible on Fig. 1b). This leads to increased reductions in the total bacterial population, and we see the Ab-sensitive bacteria decline sharply (from ~10^6^ CFU L^−1^ to ~10^4^ CFU L^−1^; Fig. 1a) while the resistant populations are less affected by the increased metal concentration, leading to an increased resistant proportion of the bacterial population, especially those resistant to copper and zinc (from ~0.5% to ~20%; Fig. 1b).

The third time scale of fluctuations within the slurry tank is associated with the 60-day cycle of emptying of the tank. The concentration of all bacterial populations increases each time the tank is emptied (because the fresh input is relatively more concentrated) before the concentrations return to the ‘continuous steady state’. However, the emptying of the slurry tank has a less pronounced effect on the bacterial populations than the effects of adding metal footbaths. None of these observed fluctuations in bacterial dynamics appear to be associated with the effluent flushing of the scraper channels every 28 days.

Fluctuations in the antibiotic resistant bacteria (both oxytetracycline and cefalexin) in the slurry tank are over approximately a single order of magnitude; this is greater than the ~2-fold fluctuations observed in the slurry-tank only model^23^, and is to be expected given the additional sources of variability in this model. However, despite this, the fluctuations of cefalexin resistant bacteria in these simulations are considerably smaller than those observed empirically^23^. Specifically, experimental sampling of the slurry tank found ESCR-EC counts with spikes from below detection threshold up to a maximum (on any single replicate) of 1.5 ×10^4^ CFU L^−1^, while simulations of our farm flow model only suggests increases in ESCR-ECs up to a maximum of approximately 3 ×10^3^ CFU L^−1^. In order to identify possible sources of this discrepancy, we carried out a global sensitivity analysis of the model’s continuous process parameters.

### Concentrations of antimicrobial-resistant bacteria were most sensitive to fitness cost and baseline death rate

**Fig. 2:**
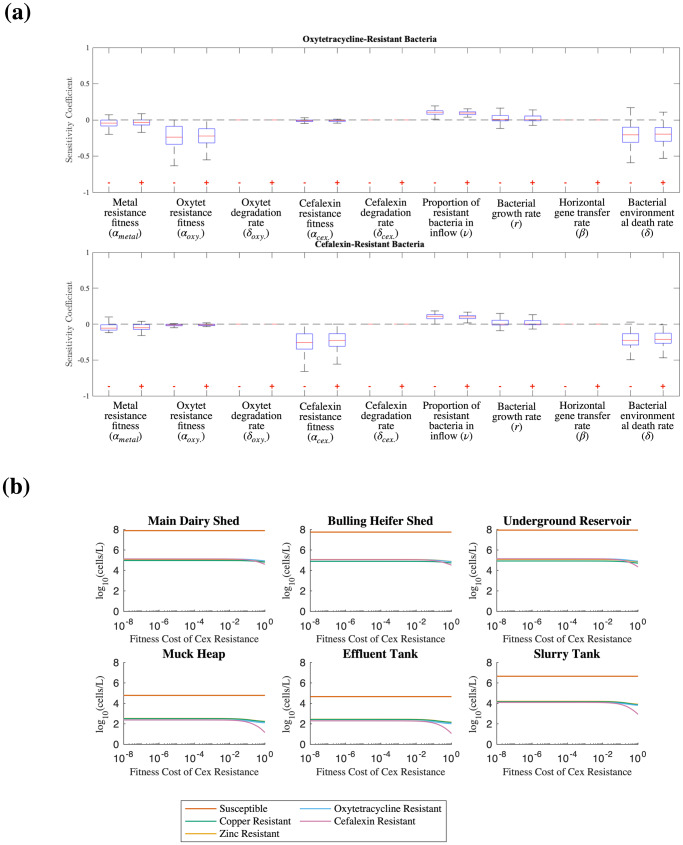
Global sensitivity analysis of bacterial parameters. **a** Boxplots of the relative sensitivity of the time-averaged oxytetracycline- and cefalexin resistant bacterial populations in the slurry tank to a change (−1% on the left, +1% on the right) in each key bacterial parameter. Each resistant population is most sensitive to the fitness cost associated with plasmid-borne resistance (α_i_), and the bacterial death rate due to environmental factors such as temperature, pH, or predation (δ). Conversely, the sensitivity coefficients for the degradation rate of both antibiotics (δ_Oxy_) and (δ_Cex_) and the rate of horizontal gene transfer (β) are negligible for both resistant bacterial populations. **b** Single parameter sensitivity analysis of the maximal spike bacterial concentrations as a function of fitness cost of cefalexin resistance. The peak concentration of cefalexin resistant *E. coli* cells in the slurry tank decreases with increasing fitness cost.

We conducted a global sensitivity analysis to determine those parameters that most affect the concentration of resistant bacteria across the farm in our model (Fig. 2a). The antimicrobial-resistant bacterial population levels were most sensitive to the fitness cost of plasmid-borne resistance carriage (α_j_) and the environmental bacterial death rate (δ), the baseline death rate without metals or antibiotics. The average concentration of resistant bacteria was also shown to be sensitive to the proportion of resistant bacteria (ν) entering the farm flow system in the heifer waste in the main dairy and bulling heifer sheds and also to the bacterial growth rate (r).

### Spikes in ESCR-ECs are consistent with chromosomal carriage of cefalexin resistance genes

The global sensitivity analysis identified resistance levels as most sensitive to the fitness cost of plasmid-borne resistance carriage. A single parameter variation analysis of the maximum bacterial populations as a function of this parameter (Fig. 2b) indicated that the spike concentration of cefalexin-resistant bacteria only reached the experimentally observed maximum levels in the periodic spikes when the fitness cost was below 10^−2^, lower than would be expected for plasmid-borne carriage. We therefore simulated the scenario where cefalexin resistance was encoded on the chromosome instead of the plasmid, in order to represent minimal fitness cost to the host.

**Fig. 3:**
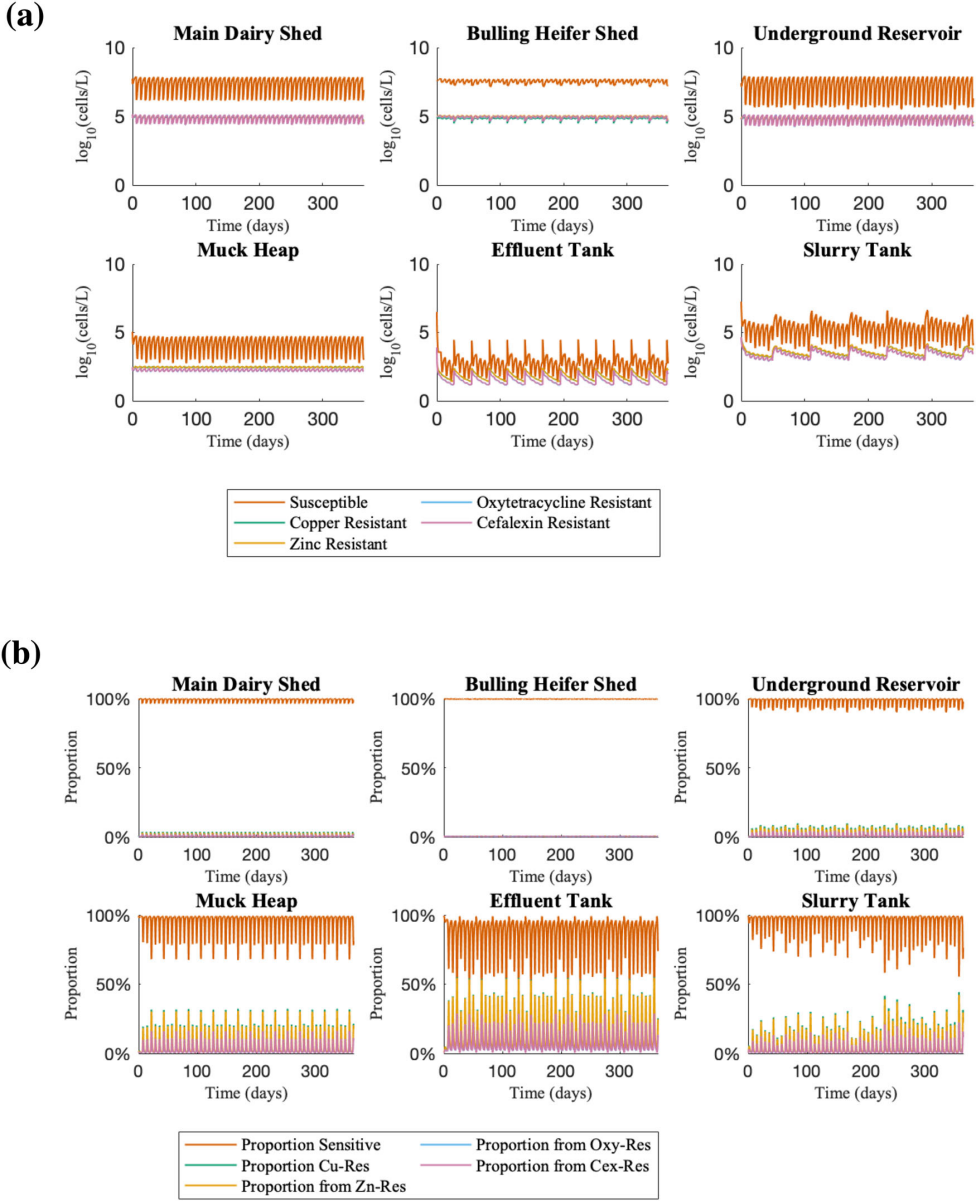
Farm flow model variant with chromosomal cefalexin-resistance. Equations can be found in Supplementary Material equations B1–B17 and a schematic as Supplementary Fig. 1. **a** Time course simulation of farm flow model where Cex-resistance is chromosomally-encoded. This time course uses the same model parameters as in the simulation where cefalexin-resistance is plasmid-encoded. Key to note are the higher spikes in cefalexin-resistant bacteria in the slurry tank as compared with the plasmid model. **b** The same time course, plotting the proportions of each sub-population (sensitive, Cu-, Zn-, Oxy- and Cex-resistant bacteria).

In simulations of chromosomal cefalexin-resistance carriage (Fig. 3), the temporal dynamics were similar to the plasmid-encoded carriage (Fig. 1). However, the amplitudes of the spikes in cefalexin-resistant populations due to periodic footbath emptying were double for chromosomally encoded resistance (fluctuations from 0.2% to 21%) compared with plasmid-encoded (0.1% to 11.9%). These concentrations are more consistent with the observed levels of spikes in ESCR-ECs from the slurry tank.

To validate these results, we analysed the *ampC* genes of the 31 sequenced *E. coli* genomes from Baker et al. ^23^, as cephalosporin resistance conferred by *ampC* mutations would necessarily be chromosomally encoded resistances. Of the 31 sequenced genomes (Supplementary Tables 9 and 10), 30 are annotated as ESCR. There are five chromosomal *ampC* variants: 14 strains are Variant 0 (wild type); 4 strains, including the non-ESCR-strain, are Variant 1, with the coding sequence mutation 70(C->T); 8 strains are Variant 2 with promoter sequence mutations -18(G->A), -1(C->T) and coding sequence mutation 58(C->T); 4 strains are Variant 3 which are similar to Variant 2 with the additional promoter mutation -42(C->T); and 1 strain is Variant 4 with the coding sequence mutations 22(C->T), 26(T->G), 27(A->T) and 32(G->A). Thus in total approximately half of these ESCR strains contain chromosomal *ampC* mutations.

### Resistant bacterial populations are insensitive to effluent flushing but highly sensitive to metal footbath emptying

**Fig. 4:**
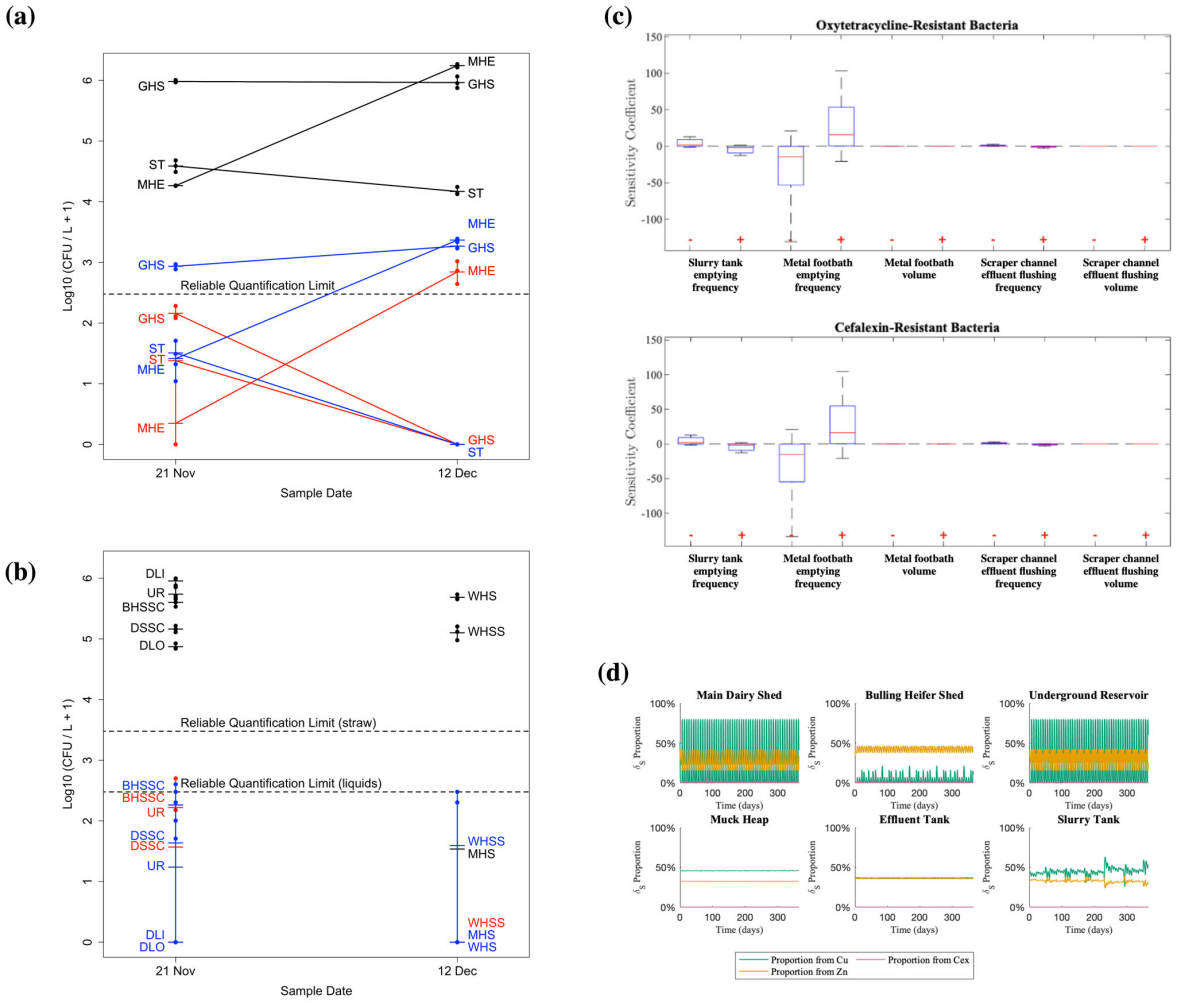
Analysis of factors potentially explaining periodic spikes in ESBL-producing *E. coli*. **a** Microbial count data from the three parts of the farm for which data were measured on both 21^st^ November and 12^th^ December: the Slurry Tank (ST), Muck Heap Effluent (MHE) and Growing Heifer Shed (GHS). Black are total *E. coli* counts as grown on TBX plates; red are *E. coli* counts grown in TBX/CTX plates; blue are *E. coli* counts as grown on ChromeAgar ESBL plates. **b** *E. coli* count data for those locations sampled on only one of the two dates: Dairy Lane Indoors (DLI), Dairy Lane Outdoors (DLO), Dairy Shed Scraper Channel (DSSC), Bulling Heifer Shed Scraper Channel (BHSSC), Underground Reservoir (UR), Muck Heap Straw (MHS), Weaned Heifer Shed (WHS), Weaned Heifer Shed Straw (WHSS); colouring by plate types is the same as **a**. **c** Global sensitivity analysis of the discrete farm management parameters. Boxplots of the relative sensitivity of the average oxytetracycline- and cefalexin resistant bacterial populations in the slurry tank to a ±1% change in key parameters for the discrete farm processes. The system is extremely sensitive to the frequency of emptying of metal footbaths. Negative sensitivity was seen to the frequency of slurry tank emptying. **d** The proportion of the death rate of sensitive bacteria δ_S_ over the time period associated with each bactericidal antimicrobial.

Two different farm activities were hypothesised to explain the regular reappearance of ESCR-producing *E. coli* in the slurry tank: periodic emptying of the metal footbaths into the slurry tank, leading to possible co-selection of ESCR-ECs by copper and zinc; and periodic flushing of the scraper channels with muck heap effluent, leading to possible re-seeding of the farm with ESCR-ECs derived from the muck heap. Support for the latter hypothesis was given by microbial counts of *E. coli* cells grown on TBX/CTX media in different farm locations (Fig. 4a, b). On 21st November, ESCR-EC strains were detected in most locations tested. On 12th December, ESCR-ECs were not detected in the slurry tank, and were only detected in the heifer sheds and, importantly, the muck heap straw. From 8th December, ambient temperatures had been below 2 °C, and the temperature was −0.5 °C at the time of sampling. While we anticipate that ESCR-ECs can be long term residents of the cattle gut, the muck heap is the one part of the external farm environment with mesophilic temperatures comparable to cattle gut temperature, due to microbial activity in the muck-heap, and so it is a reasonable hypothesis that use of muck heap effluent to flush through the scraper channels and consequently the whole slurry handling system could lead to spread of ESCR-ECs to other parts of the farm.

However, a global sensitivity analysis of the discrete farm management practice parameters (Fig. 4c) suggests that it may be the metal containing footbath use that is responsible for spikes of ESCR-ECs rather than the use of muck heap effluent. The long-term average levels of antimicrobial resistance around the farm are extremely sensitive to the metal footbath emptying frequency, and not sensitive to the scraper channel effluent flushing frequency, nor to the volumes used for the metal footbath or effluent flushing.

**Fig. 5:**
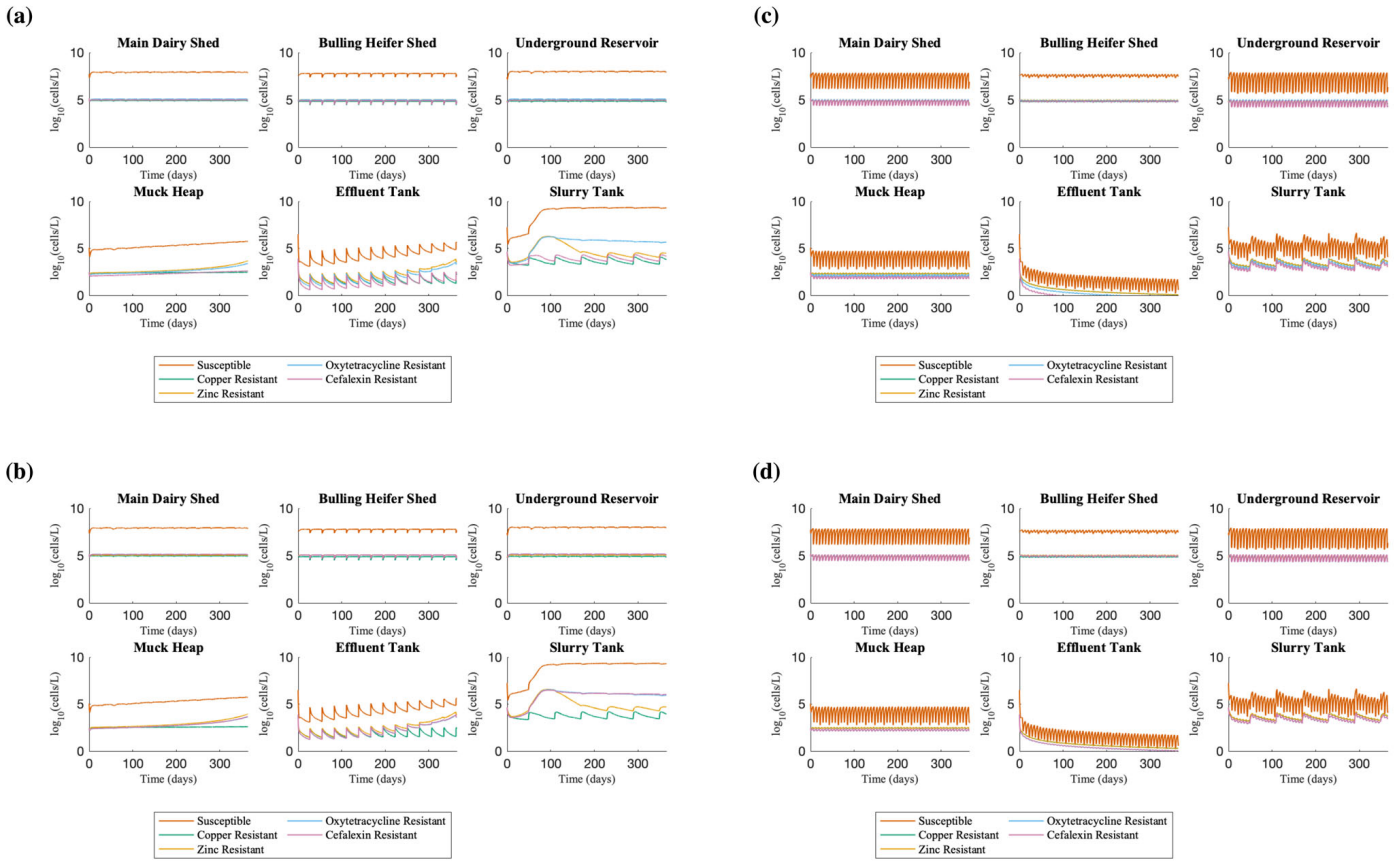
Time courses of counterfactual cases of the farm flow model. **a** Model without footbath emptying with plasmid-encoded cefalexin resistance. **b** Model without footbath emptying with cefalexin resistance encoded on the chromosome. In both **a** and **b**, the removal of the metal footbaths from the system results in a reduction in the proportion of resistance, but also a substantial increase in the total bacterial concentration to over 10^9^ CFU L^−1^. **c** Model without muck heap effluent recycling with plasmid-encoded cefalexin resistance. **d** Model without muck heap effluent recycling with cefalexin resistance encoded on the chromosome. These models show similar behaviour to the standard model (Fig. 3).

**Fig. 6:**
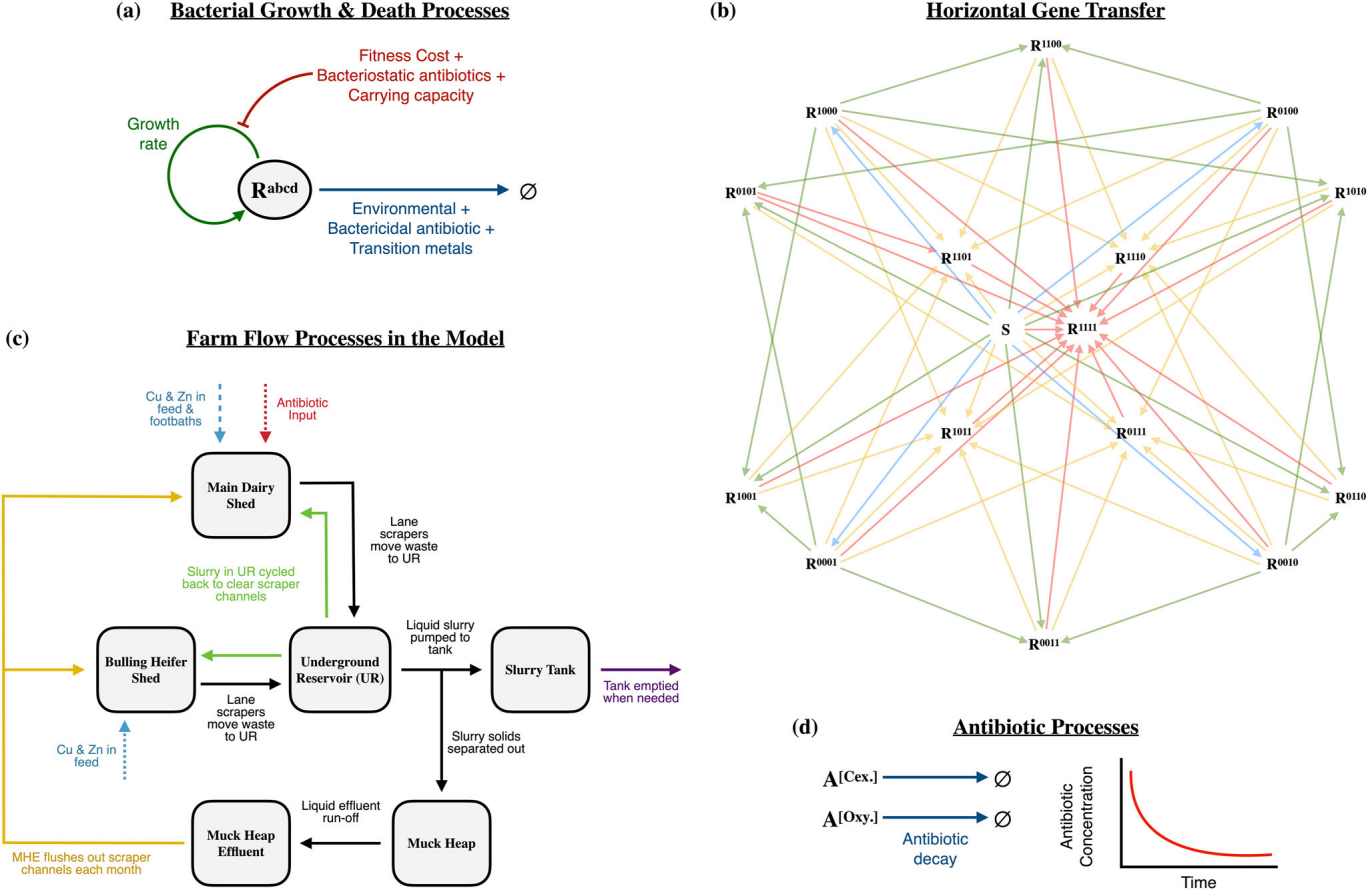
Schematic diagrams of the processes included in the farm flow model. **a** Bacterial growth and death processes, that include impacts of bacteriostatic antibiotics, toxic metals and bactericidal antibiotics. **b** Horizontal gene transfer pathways for resistance to spread between different bacterial sub-populations. S is the sensitive strain and R^xxxx^ refers to strains resistant/sensitive to differing combinations of the four antimicrobials (0 for sensitive 1 for resistance). Blue arrows show sensitive bacteria acquiring a single resistance gene, green and yellow arrows indicate the paths where bacteria become resistant to 2 or 3 antimicrobials, respectively, and red arrows indicate bacteria acquiring resistance to all 4 antimicrobials considered in this study. **c** Waste flows between the different compartments of the dairy farm that are included into the model, including farm flows (black arrows), slurry spreading to field (purple arrow), metals (blue arrows), antibiotics (red arrows), slurry recycling (green arrows) and muck heap effluent use (yellow arrows). The youngstock heifer shed (from which we present some microbial count data) is not included as it is not part of the model. **d** Antibiotic processes: antibiotics decay according to first order mass action kinetics.

To confirm that footbath emptying rather than the muck heap effluent reuse was responsible for spikes in ESCR-ECs, we ran two sets of counterfactual simulations: first, simulations without footbath being emptied into the slurry tank; second, simulations without muck heap effluent recycling. In simulations without footbath emptying, the proportion of the total bacterial population carrying resistance was typically 95% lower than in the standard model simulations, whether cefalexin resistance was carried on plasmids or chromosomes. With plasmid carriage, oxytetracycline- and cefalexin-resistant sub-populations on average consisted of 0.08% and 0.03% of the total bacterial load compared to 1.98% and 1.19% in the model with footbath emptying, while with chromosomal carriage, these sub-populations both made up on average 0.14% of the total *E. coli* population as compared to 2.65% and 2.67% in the full model. However, despite significantly lower proportions of resistance in this counterfactual scenario, the total bacterial load across the farm was considerably higher, rising to ~10^9^ CFU L^-1^ compared to ~10^6^ CFU L^−1^ (Fig. 6a, b). Moreover, oxytetracycline resistant populations also rise to ~10^6^ CFU L^−1^, because of the absence of selective pressure from Cu and Zn. When cefalexin resistance was chromosomally encoded, we also observed a sustained increased concentration level of ~10^6^ CFU L^−1^; however, in the case where cefalexin resistance is plasmid-mediated, the concentration of cefalexin-resistant bacteria in the slurry tank varies between 5 ×10^3^ and 2 ×10^4^ CFU L^−1^, only slightly higher than the maximum reached in the standard model of 6 ×10^3^ CFU L^−1^. In counterfactual simulations without muck heap effluent recycling, the outcomes are broadly similar to the standard model simulations (Fig. 5c, d), confirming that this action has minimal impact.

## Discussion

The farm flow model we have developed uses a multiscale modelling approach that generates behaviours not captured by homogeneous approaches. Many mathematical models considering AMR in agricultural settings have focussed on a within-host model^36,37^ or on a particular area of the farm, e.g. cattle shed^38^ or slurry tank^23,33,34^. While within-host models do provide scope to consider the effects of farm management on AMR, for example antimicrobial usage^35^ or the effectiveness of sequestering animals undergoing treatment^37^, it is not practical for these models to assess the effects of structural farm management practices. Other models considering the levels of AMR in single farm compartments can provide useful analysis of farm management such as the role of water troughs in maintaining bacterial loads in cattle pens^38^ or how altering slurry storage time^33,34^ or the use of a two-tank slurry storage system^23^ may control spread of resistance in dairy slurry. However, such models may not capture salient effects of practices in other areas of the farm. Thus, multiscale modelling that considers the wider farm layout could be an important modelling tool in future work considering how farm practices may affect bacterial dynamics and the spread of resistance. While our farm flow model is designed to model the layout of the particular farm considered in this study, the use of sensitivity analyses provides generality by considering a wide range of realistic farm parameters, while counterfactual analyses consider alternative farm practices. Moreover, the model could be readily adapted to the layout and waste management practices of other farms by the adjustment of the farm specific parameters and introducing (or removing) compartments dependent on the physical infrastructure. This flexible modelling approach allows for testing of farm changes on AMR outcomes that would be a serious challenge to assess empirically.

Given the impact of emptying of the transition metal containing footbaths on AMR dynamics in this system, one may consider the simplest solution would be to stop emptying transition metal containing footbaths into the main dairy shed scraper lanes. However, counterfactual simulations of the farm flow model demonstrated that while this may reduce the proportion of antibiotic resistance within the slurry flow, the absence of the repeated pulses of metals results in an overall 1000 fold higher bacterial population, and a greater and more sustained concentration of the oxytetracycline- and cefalexin-resistant *E. coli* populations. The increased bacterial load appears to be a result of copper and zinc resistances no longer being necessary for these resistant bacterial populations to survive, as the bacterial cells are no longer exposed to high levels of antimicrobial metals from the footbaths, and hence the fitness cost for carrying these genes is no longer outweighed by the excess presence of copper and zinc from the metal footbaths. That said, our previous work showed that slurry storage in the absence of fresh input leads to decreased overall and beta-lactam resistant populations of *E. coli* and other relevant species^23^; tetracycline resistance is linked to its environmental stability, implicating the importance of avoiding use of environmentally stable antibiotics if medically possible. Thus a combination of alternative metal disposal with slurry storage has the potential to lead to reduction in both the proportion and absolute abundance of cephalosporin resistant bacteria.

This leads to the question of how to dispose the waste footbath without emptying into the slurry system. It cannot be allowed to run-off into the environment as the elevated levels of copper can have a toxic impact on the environment potentially impacting on crops, vegetation and wildlife. Similarly, emptying footbath into local sewer systems if available would not be an acceptable solution as this may lead to co-selection for resistance in the sewer community, impairment of aerobic and anaerobic treatment processes in waste water treatment plants (WWTPs), and contamination of the public drinking water supply if WWTPs are unable to suitably remove the elevated transition metal levels. In the UK it is possible to remove waste footbath through a licensed contractor, but this solution is likely to be very expensive in the long term. This could prompt the suggestion that metal footbaths should not be used, but metal footbaths are commonly used in dairy farms across the UK to prevent digital dermatitis, an issue that causes 20–25% of lameness in cattle^39^, so an alternative would need to be found. Formalin footbaths are also available as an option, however, this may present other issues, as formalin is listed as a Known Human Carcinogen (KHC)^40^. Another option would be to continue using metal footbath but to recover copper and zinc from the footbaths prior to emptying using adsorbents^41^, however, such a solution may not be practical or affordable on a farm scale and may have equally surprising consequences as observed in our counter-factual simulations.

While the model suggested a substantial impact of the disposal of footbaths into the waste flow, analysis of the model also suggested that the feedback loops in the farm slurry system due to recycling of muck heap effluent to clear scraper channels had a negligible impact on the AMR profile on the farm. However, the importance of the muck heap and effluent run-off from it should not necessarily be discounted because antibiotic residues within the muck heap solids have been identified including some that have not been used on the farm for several years^23^. As farms may pivot away from the usage of antibiotics critical or important for human use, the long-term retention of resistance within the muck heap, acting like an archive of antibiotics historically used on the farm and bacterial strains carrying resistance to them, may be problematic as it may lead to the co-selection and accumulation of resistances to different antibiotics.

We have also shown the importance of considering whether ARGs are plasmid-borne or chromosomally encoded. Observed spikes in ESCR resistance across different areas of the farm were not reflected in our single-compartment model of the slurry tank^23^. In contrast, time course simulations of our initial farm flow model showed variation in the concentration of cefalexin resistance in the slurry tank, but the maximum increase observed in the plasmid-encoded case was well below the experimentally observed increase in the slurry tank. However, when we altered the model to consider a scenario where cefalexin resistance genes are encoded chromosomally, it predicted spikes in the concentration of all resistant populations corresponding to the emptying of additional metal footbaths every 3 weeks, with magnitudes more consistent with the concentrations of ESCR-ECs observed in the presumptive *E. coli* counts of the slurry. This result is consistent with the reanalysis of the *ampC* regions of our previously reported sequenced ESCR *E. coli* strains from the same farm and sampling period: 16/30 of these strains contained chromosomal mutations in either the *ampC* promoter or the coding region or both. However, these mutations would not necessarily provide cephalosporin resistance: our Variants 1–4 are close (but not identical) to Variants 15, 11, 1 and 12 of Peter Getzlaff et al. ^42^ respectively, who measured AmpC overexpression for their variants. Only (our) variant 3 displayed consistent phenotypic AmpC overexpression (6/6 of their strains); variants 1, 2 and 4 displayed AmpC overexpression in 2/8, 2/5 and 2/8 strains respectively. Moreover the phenotypic resistance patterns of our strains were highly varied: a more detailed study of the precise mechanisms of cephalosporin resistance in these ESCR strains would be warranted.

Most models of antimicrobial resistance consider the spread of resistance via conjugative plasmids^13,23,33,36,38^, and while some models have considered other mechanisms of HGT such as transduction^43,44^, these models still consider ARGs located on extra-chromosomal mobilisable elements, with an associated fitness cost of carriage^45^. The fitness cost associated with extra-chromosomal carriage is consistently a highly sensitive parameter in these models. By comparison, the sensitivity analysis also showed that the average concentrations of both oxytetracycline- and cefalexin-resistant bacteria were not sensitive to variation in the rate of horizontal gene transfer. This contrasts with our earlier slurry tank model^33^ but is consistent with our later models considering metal co-selection^13^. Metagenomic analysis of samples from the slurry tank have revealed multiple metal resistance genes (MRGs) present: *cop*, *cus*, *pco/sil* which can confer copper resistance, *czc* which can confer resistance to zinc (as well as cadmium and cobalt), as well as *mer* (mercury), *ars* (arsenic and antimony), *pbr* (lead) and *cad* (cadmium)^23^. Both *pco/sil* and *czc* genes are typically plasmid-borne^46,47^, hence our modelling assumption that both copper and zinc resistance are plasmid-mediated is reasonable. However, we only know that these *pco/sil* and *czc* genes are present in the slurry tank and not whether they are associated with *E. coli* plasmids.

Whether ARGs are located on the chromosome or on plasmids has important consequences for the risk that resistant bacteria within the slurry may pose to environmental and human health. One of the biggest risk factors of AMR within dairy slurry is transmission of ARGs into the environment by slurry spreading: the potential for ARGs to transfer cross-species, to potentially pathogenic bacteria, provides indirect pathways to impact on human health. Plasmid-borne resistance genes therefore present a greater threat within this context given the greater possibility of transmission via conjugation, while chromosomally encoded resistances may present less risk^48^ in this regard as ARGs would need an additional mobilising step before being transferred horizontally to other bacteria.

The modelling described in this paper employs an ambitious multiscale model. Great care has been taken to carefully calibrate both the core microbial model and the farm flow elements of the model, and to base the model on detailed data. However, real-world microbial communities are highly complex, with very many different species, many resistance genes to wide ranging antimicrobials, and many mobilisable elements with different transfer properties: our model, complex as it is, necessarily abstracts from that, and so is a necessarily limited description of reality. Moreover, the ODE formulation assumes well-mixed microbial communities, when in truth many microbes will live on biofilms on particulate matter, farm surfaces, walls of pipes etc. All of these factors may be important, and their rigorous evaluation would be warranted. Thus all model outcomes need empirical assessment, as they remain predictions. Our sequenced *E. coli* strains show high levels of *ampC* mutations consistent with the model predictions. This is an encouraging outcome, despite the relatively small number of strains sequenced.

A second kind of limitation is the apparent discordance between measurement of ESCR phenotypes in our previous work^23^, use of ESBL-selective media in this work, consistent with WHO recommendation of using ESBL-ECs as a sentinel for AMR^25^, and the use of the first generation cephalexin in the model, because that is the antibiotic that was used on the farm. These are all connected by the implicit assumption of cross-resistance, whether by chromosomal AmpC expression, or mobilisable beta-lactamase genes. While this is reasonable, it is recognised that each beta-lactamase gene, as well as its mutants, confers resistance to both overlapping and different sets of cephalosporin antibiotics. This is another manifestation of the complexity of AMR in the real world, and the need to make rational choices both in empirical measurements and mathematical models.

In conclusion, we have developed a hybrid discrete-continuous multiscale mathematical model of the dynamics of antimicrobial resistant bacteria within the flow of slurry around a typical high performance UK dairy farm. We have evaluated the impact of farm management practices, identified through ethnographic research, on the emergence and spread of AMR around the farm. Disposal of copper sulphate / zinc oxide footbaths into the waste flow was predicted to have a substantial effect on AMR within bacterial communities of the slurry tank. Weekly emptying of the footbaths provided periodic bactericidal inputs (particularly due to copper concentrations well in excess of the MIC) which gave rise to high magnitude fluctuations across all bacterial sub-populations modelled. The observed magnitude of fluctuations in ESCR-Ecs were predicted to occur when genes for ESCR phenotypes were chromosomally carried, consistent with genome sequencing of bacteria from the slurry. Thus we show that farm scale human practices can have a material impact on the molecular genetics of antimicrobial resistance carriage and transmission. Specifically, ESCR-Ecs could be greatly reduced through a combination of suitable and safe removal or recycling of copper and zinc from farm waste, together with prolonged slurry storage without fresh input.

## Methods

### Dairy farm background

The study considers a mid-sized, high performance commercial dairy farm in the East Midlands, UK, housing ~200 milking Holstein Friesian cattle at the time of study. Milking cattle are housed indoors in cubicle housing with concrete passageways surfaced with rubber matting, and all excreta are regularly removed from passageways by automatic scrapers into a drainage system terminating at the 3 M litre slurry tank (Fig. 6). The drainage system also receives used cleaning materials and wash water, used footbath containing zinc and copper, waste milk, and rainwater runoff. An automated screw press (Bauer S655 slurry separator with sieve size 0.75 mm; Bauer GmbH, Voitsberg, Austria) performs liquid-solid separation of the slurry tank influent. Liquids enter the slurry tank semi-continuously, while separated solids are removed to a muck heap. Weaned heifers (3–6 months) and growing heifers (6–12 months) are loose housed on straw bedding separately from milking cows; bulling heifers (12–15 months) are also housed similarly to but separately from milking cows; and individual loose box housing is also available for post-calving or sick cows. Faeces and urine from calves drain into the common drainage system, whilst dirty straw from the loose housing is taken directly to the muck heap. Excess slurry can be pumped to an 8 M litre lagoon for long term storage. Slurry is used to fertilise grassland and arable fields. Practice at this farm is typical of management methods at high-performance dairy farms, although all farms vary.

The majority of veterinary antibiotics used on the farm are aminocoumarins, aminoglycosides, beta-lactams and tetracyclines (Table S3 of Baker et al.^23^). The last recorded use of first generation cephalosporins (cephalexin) was in April 2017 (shortly before the start of the sampling period); of third generation cephalosporins (ceftiofur) was in January 2016; and of fourth generation cephalosporins (cefquinome) was in August 2015. This pattern of Ab use motivates our interest in ESCR-ECs on the farm.

### Anthropological methods

The ethnographic research^49^ was conducted over a four-month period beginning September 2017. It involved two weeks of continuous on-farm participant-observations shadowing farm staff through their daily routines. Following this, the farm was visited regularly throughout the remaining period for short engagements (one to two days or half days) to observe specific re-occurring practices of interest and in response to events of interest arising on the farm. All members of staff were shadowed at different times over this period. Observations focused on the farm staff’s everyday practices of animal management, animal disease diagnosis and treatment, and waste management. Further detail on the method and the broader ethnographic findings are reported in Helliwell et al.^50,51^.

### Mathematical model development

We have developed a mathematical model (Equations A1-A40 as Supplementary Text 1) to evaluate the risk of the spread of AMR across bacterial populations within wastewater as it flows around different areas of the farm (Fig. 6), using a multiscale, hybrid discrete-continuous, compartmental system of ordinary differential equations (ODEs).

The microbiological core of the model is a subset of the model previously described^23^, with four rather than six antimicrobials (copper, zinc, oxytetracycline and cefalexin). The core microbiological model was then embedded into each of the six compartments of the farm flow model (described below). We have chosen cefalexin as the most recently used cephalosporin on the farm, with use of human critical 3rd/4th generation cephalosporins having been discontinued. Tetracycline was included not only because of its use, but because its chemical stability leads to long term presence on the farm, with associated selection pressure leading to relatively stable high levels of tetracycline resistance, with strong accordance between the previous model and experimental data^23^. Zinc and copper were included because we are explicitly assessing the use of the zinc and copper footbath in the slurry system with the model.

Thus the bacterial resistance transfer model describes populations of antimicrobial sensitive (S_i_) and resistant (R^x1,x2,x3,x4^_i_) bacteria in each of the six compartments, where x1, x2, x3 and x4 are either 0 or 1, with x1 = 1 if the population is resistant to copper and x1 = 0 if it is sensitive to copper, and similarly x2, x3 and x4 reflect zinc, oxytetracycline and cefalexin resistant bacteria. The model includes bacterial growth (logistic equation), death (first order) and impact of bacteriostatic antibiotic on growth and bactericidal antibiotics as well as metals on death; in this way different strains may be selected for depending on antibiotic/metal concentrations. The model also includes horizontal gene transfer of resistance genes, including coupled transfer of multiple resistances on the same plasmid. We modelled the antibiotic input a^j^(t) for j ∈ {Oxy, Cex} as a discrete time-dependent parameter based on the farm antibiotic usage records for the period 1^st^ January 2017 to 31^st^ December 2017 and antibiotic degradation using first order degradation kinetics. All the parameters of the model are described in Supplementary Tables 1–6 with realistic value ranges for each parameter based either on farm observations or published literature.

We also considered a variation of the model where cefalexin-resistance (representing ESC-R) was chromosomally-encoded, with no fitness cost for cefalexin-resistance α_Cex_ and cefalexin-resistance genes only being transmitted vertically (although resistance to oxytetracycline, zinc and copper can still be spread via HGT). This is not to preclude horizontal transfer of cefalexin resistance, but rather to have a comparator model that takes the logically extreme position of only vertical transfer, recognising that the real environment will contain a mix of the two. The variant of the farm flow model with chromosomal cefalexin-resistance can be found in Supplementary Text 2 as equations B1–B17, with the structure of resistance transfer illustrated in Supplementary Fig. 1.

The six different farm compartments (Fig. 6) are described by a volumetric flow ODE model, to describe the flow of dirty water (V_i_) between the compartments, in which the rates of flow between the different compartments follow first order mass action kinetics, and materials within the waste (including the microbial strains of the core model described above) flow between compartments with the liquid in which they are dissolved/suspended. The farm flow compartments were defined through pre-existing knowledge of the dairy farm, as well as participant observations from the anthropological work. The ethnographic participant observations identified additional waste management infrastructure and practices that resulted in two feedback loops within the system that had not been previously identified through discussions with farm management. These feedback loops are identified via the yellow and green arrows on the farm flow diagram (Fig. 6).

The flow model was then extended to include the concentrations of copper and zinc (M^Cu^_i_ and M^Zn^_i_), and antibiotics (A^Oxy^_i_ and A^Cex^_i_). We assume that the volume of daily waste inputs in the main dairy shed (a) and bulling heifer shed (b) (i.e., from faecal matter, trough water, footbaths, bedding etc.) are constant. Copper and zinc are used in the cattle feed as standard mineral supplementation. The majority of these metals are not absorbed by the cow (~99% and 85% are excreted for Cu and Zn respectively^52^) and enter the slurry flow system from faeces. We also assume that the solid slurry matter separated onto the muck heap has minimal residual liquid so effluent run off is determined only by rainfall (η).

In addition to the continuous flow model, three farm processes, reflective of actual on-farm practice are represented by discrete processes: the emptying of metal containing footbaths into the main dairy shed scraper channel; the flushing of the scraper channels with muck heap effluent; and the emptying of the slurry tank. To model these processes, the volume of the footbath V_footbath_ and mass of copper and zinc (a^Cu^_footbath_ and a^Zn^_footbath_) are added to the slurry volume and metal mass in the main dairy shed respectively (the scraper channels are not distinguished from the overall shed in the model) at regular time intervals given by T_footbath_. The emptying of the slurry tank is modelled similarly with time intervals T_tank_. We model the tank as not being completely emptied and that a small proportion (0 < ε_tank_ « 1) of the volume, mass of metals and antibiotics and population of bacteria in the slurry tank contents remains. Similarly, the muck heap effluent tank is emptied with time intervals T_Eff. flush_; this is used to wash out the scraper channels, so in the model most enters the tank, a small fraction (0 < ε_tank_ « 1) remains and (1 − ε_eff_)V_eff_/2 is added to the main dairy and bulling heifer sheds (where the scraper channels are located).

### Simulations

We simulated our farm flow model using MATLAB R2020^53^. We produced time course simulations with the standard parameter values (Supplementary Tables 1–6) using the ODE45 solver to show the concentration of the different bacterial populations over time. For all simulations of the model, we used the steady state values of the continuous farm flow model (i.e. from a long simulation of the model without discrete processes) for the initial conditions of the slurry volume and metal equations, and assume that the initial volume of slurry in the tank is 10^6 ^L and that the effluent tank has recently been used and is thus nearly empty (V_eff_ = ω) to avoid division by zero errors in the HGT terms of the effluent bacterial populations. We initialised the bacterial populations in our model using the average *E. coli* counts sampled from each area of the farm and assumed the proportion of each distinct resistant bacterial population was the same. Code for the two variants of the model is provided as Supplementary Code 1 and 2.

### Sensitivity analysis

We performed a global sensitivity analysis of bacterial parameters and discrete farm practice parameters to determine what factors have the most influence on the concentration of oxytetracycline- and cefalexin-resistance within the slurry tank, using Latin Hypercube Elasticity Analysis as previously described^13,33^. For each parameter, we took 1000 parameter values sampled from the feasible parameter space (Supplementary Tables 7 and 8) using Latin hypercube sampling. We then completed a local one-at-a-time elasticity analysis for each parameter value. Sensitivity analyses were also carried out in Matlab R2020.

### Microbiological sampling

Liquid samples were collected from different areas on the farm on two different dates. On 21st November 2017, samples were taken from the main dairy cubicle shed (dairy lane inside, dairy lane outside the scraper channel), bulling heifer cubicle shed and scraper channel, underground reservoir, the muck heap effluent tank and the slurry tank. On 12th December 2017, samples were taken from the bulling heifer shed (as before), the sheds containing weaned and growing heifers (not in Fig. 6), straw from the weaned heifer shed, muck heap straw, the muck heap effluent tank and the slurry tank. *E. coli* strains were isolated using Tryptone Bile X-Glucuronide (TBX) or MacConkey agar or TBX/MacConkey supplemented with 16 μg ml^-1^ ampicillin (AMP), or 2 μg ml^-1^ cefotaxime (CTX); or on CHROMagar ESBL^TM^ agar, as described previously^22^. The CTX and CHROMagar plates were specifically used because ESBL-EC are the WHO recommended indicator strains^25^. Putative *E. coli* isolates were subcultured onto TBX agar or TBX agar supplemented with 2 μg ml^−^^1^ CTX. *E. coli* strains were confirmed using oxidase and catalase tests as described^23^.

### Genome sequence analysis

We reanalysed the *ampC* regions for potential chromosomal mutations that could provide cephalosporin resistance in the 31 *E. coli* genomes sequenced as part of our previous study^23^. Twenty-five of these strains were sequenced as ESCR phenotypes, while 6 strains were sequenced because of potential mercury resistance, of which 5 were also ESCRs. All whole genome fasta files were downloaded from the European Nucleotide Archive (ENA) under the project number PRJNA736866. Each genome was parsed through IPCRESS, part of the EXONERATE (v2.2) tool package^54^, using primers 5’-GATCGTTCTGCCGCTGTG-3’ and 5’-GGGCAGCAAATGTGGAGCAA-3’ to isolate the *ampC* promotor and attenuator regions^42^. Details of strains are provided in Supplementary Table 9 (WT *ampC* strains) and Supplementary Table 10 (mutant *ampC* strains).

### Reporting summary

Further information on research design is available in the Nature Research Reporting Summary linked to this article.

### Supplementary information

Supplementary Information

Supplementary Code 1

Supplementary Code 2

Microbiology Data

Reporting Summary

## Data Availability

The microbiology count data used for Fig. 4 is supplied as Supplementary Data File 1. The Matlab code for the model with plasmid and chromosomal gene carriage are supplied as Supplementary Code 1 and 2 respectively.
