# Supplementary material for: Modelling the impact of wastewater flows and management practices on antimicrobial resistance in dairy farms
Source: NPJ Antimicrob Resist. 2024 May 14;2:13. doi: 10.1038/s44259-024-00029-4 (PMC11093733; doi:10.1038/s44259-024-00029-4)
Supplement: Supplementary file 1 — Supplementary Information [file 44259_2024_29_MOESM1_ESM.pdf]

Modelling the impact of wastewater flows and management practices on antimicrobial resistance in dairy farms: Supplementary Material

Henry Todman, Richard Helliwell, Liz King, Steven P Hooton, Michelle Baker, Jean Margerison, Paul Wilson, Christine E R Dodd, Carol Morris, Sujatha Raman, Chris Hudson, Jan-Ulrich Kreft, Jon L Hobman, Theodore Kypraios, Dov J Stekel

Supplementary Text 1: Model Equations for Plasmid-Borne Resistance

We have developed a mathematical model describing the interactions between antimicrobial resistant and sensitive bacterial populations within the flow of slurry across a typical high-performance dairy farm in the UK. This model considers 6 different areas of the farm (the main dairy shed, bulling heifer shed, underground reservoir (UR), muck heap, effluent tank and slurry tank), and in each different area we model the volume of slurry ((01)-(06)), the mass of metals within the slurry ((07)-(012)), the mass of antibiotics within the slurry ((013)-(018)) and the populations of sensitive and resistant *E.coli* ((019)-(040)), giving a system of 126 ordinary differential equations.

Volume Flow

(01)-(06)

$$\begin{aligned}\frac{dV_{\text{dairy}}}{dt} &= a - \rho V_{\text{dairy}} + \frac{\sigma V_{\text{UR}}}{2} + \Theta\left(T_{\text{footbath}}\right) V_{\text{footbath}} \\ &\quad + \Theta\left(T_{\text{extra foot.}}\right) V_{\text{extra foot.}} + \Theta\left(T_{\text{Eff. flush}}\right) \frac{\left(1-\epsilon_{\text{eff}}\right) V_{\text{eff}}}{2}\ ,\end{aligned} \tag{01}$$

$$\frac{dV_{\text{heifer}}}{dt} = b - \rho V_{\text{heifer}} + \frac{\sigma V_{\text{UR}}}{2} + \Theta\left(T_{\text{Eff. flush}}\right) \frac{\left(1-\epsilon_{\text{eff}}\right) V_{\text{eff}}}{2}\ , \tag{02}$$

$$\frac{dV_{\text{UR}}}{dt} = \rho\left(V_{\text{dairy}} + V_{\text{heifer}}\right) - \sigma V_{\text{UR}} - \gamma V_{\text{UR}}\ , \tag{03}$$

$$\frac{dV_{\text{muck}}}{dt} = \left(1-\varepsilon\right)\gamma V_{\text{UR}} - \eta V_{\text{muck}} - \kappa_{\text{muck}} V_{\text{muck}}\ , \tag{04}$$

$$\frac{dV_{\text{eff}}}{dt} = \eta V_{\text{muck}} + \iota_{\text{silage}} - \Theta\left(T_{\text{Effluent}}\right)\left(1-\epsilon_{\text{eff}}\right) V_{\text{eff}}\ , \tag{05}$$

$$\frac{dV_{\text{tank}}}{dt} = \varepsilon\gamma V_{\text{UR}} - \Theta\left(T_{\text{Tank empty}}\right)\left(1-\epsilon_{\text{Tank}}\right) V_{\text{tank}}\ , \tag{06}$$

where $\Theta(T) = \begin{cases} 1 & t \in T \\ 0 & \text{otherwise} \end{cases}$.

Metal Mass Flow

$$\begin{aligned}\frac{dM^{[j]}_{\text{dairy}}}{dt} &= a^{[j]}_{\text{feed}} - \rho M^{[j]}_{\text{dairy}} + \frac{\sigma M^{[j]}_{\text{UR}}}{2} + \Theta\left(T_{\text{footbath}}\right) a^{[j]}_{\text{footbath}} \\ &\quad + \Theta\left(T_{\text{extra foot.}}\right) a^{[j]}_{\text{extra foot.}} + \Theta\left(T_{\text{Eff. flush}}\right) \frac{\left(1-\epsilon_{\text{eff}}\right) M^{[j]}_{\text{eff}}}{2}\ ,,\end{aligned} \tag{07}$$

$$\frac{dM^{[j]}_{\text{heifer}}}{dt} = b^{[j]}_{\text{feed}} - \rho M^{[j]}_{\text{heifer}} + \frac{\sigma M^{[j]}_{\text{UR}}}{2} + \Theta\left(T_{\text{Eff. flush}}\right) \frac{\left(1-\epsilon_{\text{eff}}\right) M^{[j]}_{\text{eff}}}{2}\ , \tag{08}$$

$$\frac{dM^{[j]}_{\text{UR}}}{dt} = \rho\left(M^{[j]}_{\text{dairy}} + M^{[j]}_{\text{heifer}}\right) - \sigma M^{[j]}_{\text{UR}} - \gamma M^{[j]}_{\text{UR}}, \tag{09}$$

$$\frac{dM^{[j]}_{\text{muck}}}{dt} = \left(1-\varepsilon\right)\gamma M^{[j]}_{\text{UR}} - \eta M^{[j]}_{\text{muck}} - \kappa_{\text{muck}} M^{[j]}_{\text{muck}}, \tag{010}$$

$$\frac{dM^{[j]}_{\text{eff}}}{dt} = \eta M^{[j]}_{\text{muck}} - \Theta\left(T_{\text{Effluent}}\right)\left(1-\epsilon_{\text{eff}}\right) M^{[j]}_{\text{eff}}\ ,, \tag{011}$$

$$\frac{dM^{[j]}_{\text{tank}}}{dt} = \varepsilon\gamma M^{[j]}_{\text{UR}} - \Theta\left(T_{\text{Tank empty}}\right)\left(1-\epsilon_{\text{Tank}}\right) M^{[j]}_{\text{tank}}\ , \tag{012}$$

where $j \in \{\text{Cu}, \text{Zn}\}$.

Antibiotic Mass Flow

$$\frac{dA_{\text{dairy}}^{[j]}}{dt} = a^{[j]}(t) - \rho A_{\text{dairy}}^{[j]} + \frac{\sigma A_{\text{UR}}^{[j]}}{2} - \delta_{[j]} A_{\text{dairy}}^{[j]} + \Theta\left(T_{\text{Eff. flush}}\right) \frac{\left(1-\epsilon_{\text{eff}}\right) A_{\text{eff}}^{[j]}}{2} \;,, \tag{013}$$

$$\frac{dA_{\text{heifer}}^{[j]}}{dt} = -\rho A_{\text{heifer}}^{[j]} + \frac{\sigma A_{\text{UR}}^{[j]}}{2} - \delta_{[j]} A_{\text{heifer}}^{[j]} + \Theta\left(T_{\text{Eff. flush}}\right) \frac{\left(1-\epsilon_{\text{eff}}\right) A_{\text{eff}}^{[j]}}{2} \;, \tag{014}$$

$$\frac{dA_{\text{UR}}^{[j]}}{dt} = \rho\left(A_{\text{dairy}}^{[j]} + A_{\text{heifer}}^{[j]}\right) - \sigma A_{\text{UR}}^{[j]} - \gamma A_{\text{UR}}^{[j]} - \delta_{[j]} A_{\text{UR}}^{[j]}, \tag{015}$$

$$\frac{dA_{\text{muck}}^{[j]}}{dt} = (1-\varepsilon)\gamma A_{\text{UR}}^{[j]} - \eta A_{\text{muck}}^{[j]} - \kappa_{\text{muck}} A_{\text{muck}}^{[j]} - \delta_{[j]} A_{\text{muck}}^{[j]}, \tag{016}$$

$$\frac{dA_{\text{eff}}^{[j]}}{dt} = \eta A_{\text{muck}}^{[j]} - \delta_{[j]} A_{\text{eff}}^{[j]} - \Theta\left(T_{\text{Effluent}}\right)\left(1-\epsilon_{\text{eff}}\right) A_{\text{eff}}^{[j]} \;,, \tag{017}$$

$$\frac{dA_{\text{tank}}^{[j]}}{dt} = \varepsilon\gamma A_{\text{UR}}^{[j]} - \delta_{[j]} A_{\text{tank}}^{[j]} - \Theta\left(T_{\text{Tank empty}}\right)\left(1-\epsilon_{\text{Tank}}\right) A_{\text{tank}}^{[j]} \;, \tag{018}$$

where $j \in \{\text{Oxy}, \text{Cex}\}$.

Bacteria

In each compartment $i \in \{\text{dairy, heifer, UR, muck, eff., tank}\}$, we shall denote bacterial populations by $R_i^{[x_1,x_2,x_3,x_4]}$, where $[x_1,x_2,x_3,x_4] \in \{0,1\}^4$ such that $x_1 = 1$ if the population is resistant to copper, while $x_1 = 0$ if it is sensitive to copper, and similarly x_2, x_3 and x_4 reflect zinc, oxytetracycline and cefalexin resistance or sensitivity respectively. We shall denote the bacterial population sensitive to all antimicrobial agents as S_i (i.e. $S_i = R_i^{[0,0,0,0]}$)

We define Ω_i as the set of all bacterial populations within the compartment i:

$$\Omega_i = \left\{S_i, R_i^{[1,0,0,0]}, R_i^{[0,1,0,0]}, \ldots, R_i^{[1,1,1,1]}\right\},$$

We shall also define Ω_i^* as the set of all bacterial populations carrying at least one resistance within the compartment i, i.e. $\Omega_i^* = \Omega_i \;/ \{S_i\}$.

We shall also describe the flow of each bacterial population, $R_i^{[x_1,x_2,x_3,x_4]}$, between the different farm areas by the function $\mathscr{F} : \Omega_i \to \mathbb{R}$ such that:

$$\mathscr{F}(R_i^{[x_1,x_2,x_3,x_4]}) = \begin{cases} a\nu_{[x_1,x_2,x_3,x_4]}\psi_{\text{E.coli}} - \rho R_{\text{dairy}}^{[x_1,x_2,x_3,x_4]} + \frac{\sigma R_{\text{UR}}^{[x_1,x_2,x_3,x_4]}}{2} + \frac{\omega R_{\text{eff}}^{[x_1,x_2,x_3,x_4]}}{2V_{\text{dairy}}}, & \text{for } i = \text{dairy} \\ b\nu_{[x_1,x_2,x_3,x_4]}\psi_{\text{E.coli}} - \rho R_{\text{heifer}}^{[x_1,x_2,x_3,x_4]} + \frac{\sigma R_{\text{UR}}^{[x_1,x_2,x_3,x_4]}}{2} + \frac{\omega R_{\text{eff}}^{[x_1,x_2,x_3,x_4]}}{2V_{\text{heifer}}}, & \text{for } i = \text{heifer} \\ \rho\left(R_{\text{dairy}}^{[x_1,x_2,x_3,x_4]} + R_{\text{heifer}}^{[x_1,x_2,x_3,x_4]}\right) - \sigma R_{\text{UR}}^{[x_1,x_2,x_3,x_4]} - \gamma R_{\text{UR}}^{[x_1,x_2,x_3,x_4]}, & \text{for } i = \text{UR} \\ (1-\varepsilon)\gamma R_{\text{UR}}^{[x_1,x_2,x_3,x_4]} - \eta R_{\text{muck}}^{[x_1,x_2,x_3,x_4]} - \kappa_{\text{muck}} R_{\text{muck}}^{[x_1,x_2,x_3,x_4]}, & \text{for } i = \text{muck} \\ \eta R_{\text{muck}}^{[x_1,x_2,x_3,x_4]} - \frac{\omega R_{\text{eff}}^{[x_1,x_2,x_3,x_4]}}{V_{\text{heifer}}}, & \text{for } i = \text{eff} \\ \varepsilon\gamma R_{\text{UR}}^{[x_1,x_2,x_3,x_4]} - \kappa_{\text{tank}} R_{\text{tank}}^{[x_1,x_2,x_3,x_4]}, & \text{for } i = \text{tank} \end{cases} \tag{019}$$

We shall also define the effect of the discrete farm processes on each bacterial population, $R_i^{[x_1,x_2,x_3,x_4]}$, by the function $\mathscr{D} : \Omega_i \to \mathbb{R}$ such that:

$$\mathscr{D}(R_i^{[x_1,x_2,x_3,x_4]}) = \begin{cases} \Theta\left(T_{\text{Eff. flush}}\right) \frac{(1-\epsilon_{\text{eff}}) R_{\text{eff}}^{[x_1,x_2,x_3,x_4]}}{2}, & \text{for } i = \text{dairy, heifer} \\ 0, & \text{for } i = \text{UR, muck} \\ -\Theta\left(T_{\text{Eff. flush}}\right)\left(1-\epsilon_{\text{eff}}\right) R_{\text{eff}}^{[x_1,x_2,x_3,x_4]}, & \text{for } i = \text{eff} \\ -\Theta\left(T_{\text{Tank empty}}\right)\left(1-\epsilon_{\text{tank}}\right) R_{\text{tank}}^{[x_1,x_2,x_3,x_4]}, & \text{for } i = \text{tank} \end{cases} \tag{020}$$

Similarly, we describe the growth and death of each bacterial population, $R_i^{[x_1,x_2,x_3,x_4]}$, by the function $\mathscr{G} : \Omega_i \to \mathbb{R}$ such that:

$$\begin{aligned}\mathscr{G}(R_i^{[x_1,x_2,x_3,x_4]}) =& rR_i^{[x_1,x_2,x_3,x_4]}\big)\left(1 - x_1\alpha_{[\mathrm{Cu}]} - x_2\alpha_{[\mathrm{Zn}]} - x_3\alpha_{[\mathrm{Oxy}]} - x_4\alpha_{[\mathrm{Cex}]}\right)\left(1 - \frac{N_{\mathrm{i}}}{N_{\max}}\right) \\ &\left(1 - (1-x_3)ES_{[\mathrm{Oxy}]}\right) - \big((1-x_1)ES_{[\mathrm{Cu}]} - (1-x_2)ES_{[\mathrm{Zn}]} - (1-x_4)ES_{[\mathrm{Cex}]} \\ &- \delta\big)R_i^{[x_1,x_2,x_3,x_4]}\end{aligned} \tag{021}$$

Where N_{i} denotes the total bacterial population in the compartment $i \in$ {dairy, heifer, UR, muck, eff, tank}, such that:

$$N_{\mathrm{i}} = \$ \sum_{\mathscr{R}_i \in \Omega_i} (\mathscr{R}_i)\,, \tag{022}$$

and $E_{\mathrm{i}}^{[j]}$ (for $i \in$ {dairy, heifer, UR, muck, eff, tank} and $j \in$ {Cu, Zn, Oxy, Cex}) denotes the antimicrobial effect on the bacterial growth rate in the case of bacteriostatic antibiotics (e.g. oxytetracycline), or the antimicrobial effect on the bacterial death rate in the case of bacteriocidal antimicrobials (e.g. copper, zinc or cefalexin).

$$E_{\mathrm{i}}^{[j]} = \frac{E_{\max}^{[j]}\left({}^{A_i}\!/_{V_i}\right)^{H_{[j]}}}{MIC_{[j]}{}^{H_{[j]}} + \left({}^{A_i}\!/_{V_i}\right)^{H_{[j]}}} \tag{023}$$

We shall also describe the horizontal transfer of resistance between bacterial populations by the function $\mathscr{H} : \Omega_i \to \mathbb{R}$ such that:

$$\begin{aligned}\mathscr{H}(S_i) =& -\beta S_i\left(\frac{R_i^{[1,0,0,0]}}{S_i + R_i^{[1,0,0,0]}} + \frac{R_i^{[0,1,0,0]}}{S_i + R_i^{[0,1,0,0]}} + \frac{R_i^{[0,0,1,0]}}{S_i + R_i^{[0,0,1,0]}} + \frac{R_i^{[0,0,0,1]}}{S_i + R_i^{[0,0,0,1]}} + \right. \\ &\frac{R_i^{[1,1,0,0]}}{S_i + R_i^{[1,1,0,0]}} + \frac{R_i^{[1,0,1,0]}}{S_i + R_i^{[1,0,1,0]}} + \frac{R_i^{[1,0,0,1]}}{S_i + R_i^{[1,0,0,1]}} + \frac{R_i^{[0,1,1,0]}}{S_i + R_i^{[0,1,1,0]}} + \\ &\frac{R_i^{[0,1,0,1]}}{S_i + R_i^{[0,1,0,1]}} + \frac{R_i^{[0,0,1,1]}}{S_i + R_i^{[0,0,1,1]}} + \frac{R_i^{[1,1,1,0]}}{S_i + R_i^{[1,1,1,0]}} + \frac{R_i^{[1,1,0,1]}}{S_i + R_i^{[1,1,0,1]}} + \\ &\left.\frac{R_i^{[1,0,1,1]}}{S_i + R_i^{[1,0,1,1]}} + \frac{R_i^{[0,1,1,1]}}{S_i + R_i^{[0,1,1,1]}} + \frac{R_i^{[1,1,1,1]}}{S_i + R_i^{[1,1,1,1]}}\right),\end{aligned} \tag{024}$$

$$\begin{aligned}\mathscr{H}\left(R_i^{[1,0,0,0]}\right) =& \frac{\beta S_i R_i^{[1,0,0,0]}}{S_i + R_i^{[1,0,0,0]}} - \beta R_i^{[1,0,0,0]}\left(\frac{R_i^{[0,1,0,0]}}{R_i^{[1,0,0,0]} + R_i^{[0,1,0,0]}} + \frac{R_i^{[0,0,1,0]}}{R_i^{[1,0,0,0]} + R_i^{[0,0,1,0]}} + \right. \\ &\frac{R_i^{[0,0,0,1]}}{R_i^{[1,0,0,0]} + R_i^{[0,0,0,1]}} + \frac{R_i^{[1,1,0,0]}}{R_i^{[1,0,0,0]} + R_i^{[1,1,0,0]}} + \frac{R_i^{[1,0,1,0]}}{R_i^{[1,0,0,0]} + R_i^{[1,0,1,0]}} + \\ &\frac{R_i^{[1,0,0,1]}}{R_i^{[1,0,0,0]} + R_i^{[1,0,0,1]}} + \frac{R_i^{[0,1,1,0]}}{R_i^{[1,0,0,0]} + R_i^{[0,1,1,0]}} + \frac{R_i^{[0,1,0,1]}}{R_i^{[1,0,0,0]} + R_i^{[0,1,0,1]}} + \\ &\frac{R_i^{[0,0,1,1]}}{R_i^{[1,0,0,0]} + R_i^{[0,0,1,1]}} + \frac{R_i^{[1,1,1,0]}}{R_i^{[1,0,0,0]} + R_i^{[1,1,1,0]}} + \frac{R_i^{[1,1,0,1]}}{R_i^{[1,0,0,0]} + R_i^{[1,1,0,1]}} + \\ &\left.\frac{R_i^{[1,0,1,1]}}{R_i^{[1,0,0,0]} + R_i^{[1,0,1,1]}} + \frac{R_i^{[0,1,1,1]}}{R_i^{[1,0,0,0]} + R_i^{[0,1,1,1]}} + \frac{R_i^{[1,1,1,1]}}{R_i^{[1,0,0,0]} + R_i^{[1,1,1,1]}}\right),\end{aligned} \tag{025}$$

$$
\begin{aligned}
\mathscr{H}\left(R_i^{[0,1,0,0]}\right) = \frac{\beta S_i R_i^{[0,1,0,0]}}{S_i + R_i^{[0,1,0,0]}} - \beta R_i^{[0,1,0,0]} \Bigg(& \frac{R_i^{[1,0,0,0]}}{R_i^{[0,1,0,0]} + R_i^{[1,0,0,0]}} + \frac{R_i^{[0,0,1,0]}}{R_i^{[0,1,0,0]} + R_i^{[0,0,1,0]}} + \\
& \frac{R_i^{[0,0,0,1]}}{R_i^{[0,1,0,0]} + R_i^{[0,0,0,1]}} + \frac{R_i^{[1,1,0,0]}}{R_i^{[0,1,0,0]} + R_i^{[1,1,0,0]}} + \frac{R_i^{[1,0,1,0]}}{R_i^{[0,1,0,0]} + R_i^{[1,0,1,0]}} + \\
& \frac{R_i^{[1,0,0,1]}}{R_i^{[0,1,0,0]} + R_i^{[1,0,0,1]}} + \frac{R_i^{[0,1,1,0]}}{R_i^{[0,1,0,0]} + R_i^{[0,1,1,0]}} + \frac{R_i^{[0,1,0,1]}}{R_i^{[0,1,0,0]} + R_i^{[0,1,0,1]}} + \\
& \frac{R_i^{[0,0,1,1]}}{R_i^{[0,1,0,0]} + R_i^{[0,0,1,1]}} + \frac{R_i^{[1,1,1,0]}}{R_i^{[0,1,0,0]} + R_i^{[1,1,1,0]}} + \frac{R_i^{[1,1,0,1]}}{R_i^{[0,1,0,0]} + R_i^{[1,1,0,1]}} + \\
& \frac{R_i^{[1,0,1,1]}}{R_i^{[0,1,0,0]} + R_i^{[1,0,1,1]}} + \frac{R_i^{[0,1,1,1]}}{R_i^{[0,1,0,0]} + R_i^{[0,1,1,1]}} + \frac{R_i^{[1,1,1,1]}}{R_i^{[0,1,0,0]} + R_i^{[1,1,1,1]}} \Bigg),
\end{aligned} \tag{026}
$$

$$
\begin{aligned}
\mathscr{H}\left(R_i^{[0,0,1,0]}\right) = \frac{\beta S_i R_i^{[0,0,1,0]}}{S_i + R_i^{[0,0,1,0]}} - \beta R_i^{[0,0,1,0]} \Bigg(& \frac{R_i^{[1,0,0,0]}}{R_i^{[0,0,1,0]} + R_i^{[1,0,0,0]}} + \frac{R_i^{[0,1,0,0]}}{R_i^{[0,0,1,0]} + R_i^{[0,1,0,0]}} + \\
& \frac{R_i^{[0,0,0,1]}}{R_i^{[0,0,1,0]} + R_i^{[0,0,0,1]}} + \frac{R_i^{[1,1,0,0]}}{R_i^{[0,0,1,0]} + R_i^{[1,1,0,0]}} + \frac{R_i^{[1,0,1,0]}}{R_i^{[0,0,1,0]} + R_i^{[1,0,1,0]}} + \\
& \frac{R_i^{[1,0,0,1]}}{R_i^{[0,0,1,0]} + R_i^{[1,0,0,1]}} + \frac{R_i^{[0,1,1,0]}}{R_i^{[0,0,1,0]} + R_i^{[0,1,1,0]}} + \frac{R_i^{[0,1,0,1]}}{R_i^{[0,0,1,0]} + R_i^{[0,1,0,1]}} + \\
& \frac{R_i^{[0,0,1,1]}}{R_i^{[0,0,1,0]} + R_i^{[0,0,1,1]}} + \frac{R_i^{[1,1,1,0]}}{R_i^{[0,0,1,0]} + R_i^{[1,1,1,0]}} + \frac{R_i^{[1,1,0,1]}}{R_i^{[0,0,1,0]} + R_i^{[1,1,0,1]}} + \\
& \frac{R_i^{[1,0,1,1]}}{R_i^{[0,0,1,0]} + R_i^{[1,0,1,1]}} + \frac{R_i^{[0,1,1,1]}}{R_i^{[0,0,1,0]} + R_i^{[0,1,1,1]}} + \frac{R_i^{[1,1,1,1]}}{R_i^{[0,0,1,0]} + R_i^{[1,1,1,1]}} \Bigg),
\end{aligned} \tag{027}
$$

$$
\begin{aligned}
\mathscr{H}\left(R_i^{[0,0,0,1]}\right) = \frac{\beta S_i R_i^{[0,0,0,1]}}{S_i + R_i^{[0,0,0,1]}} - \beta R_i^{[0,0,0,1]} \Bigg(& \frac{R_i^{[1,0,0,0]}}{R_i^{[0,0,0,1]} + R_i^{[1,0,0,0]}} + \frac{R_i^{[0,1,0,0]}}{R_i^{[0,0,0,1]} + R_i^{[0,1,0,0]}} + \\
& \frac{R_i^{[0,0,1,0]}}{R_i^{[0,0,0,1]} + R_i^{[0,0,1,0]}} + \frac{R_i^{[1,1,0,0]}}{R_i^{[0,0,0,1]} + R_i^{[1,1,0,0]}} + \frac{R_i^{[1,0,1,0]}}{R_i^{[0,0,0,1]} + R_i^{[1,0,1,0]}} + \\
& \frac{R_i^{[1,0,0,1]}}{R_i^{[0,0,0,1]} + R_i^{[1,0,0,1]}} + \frac{R_i^{[0,1,1,0]}}{R_i^{[0,0,0,1]} + R_i^{[0,1,1,0]}} + \frac{R_i^{[0,1,0,1]}}{R_i^{[0,0,0,1]} + R_i^{[0,1,0,1]}} + \\
& \frac{R_i^{[0,0,1,1]}}{R_i^{[0,0,0,1]} + R_i^{[0,0,1,1]}} + \frac{R_i^{[1,1,1,0]}}{R_i^{[0,0,0,1]} + R_i^{[1,1,1,0]}} + \frac{R_i^{[1,1,0,1]}}{R_i^{[0,0,0,1]} + R_i^{[1,1,0,1]}} + \\
& \frac{R_i^{[1,0,1,1]}}{R_i^{[0,0,0,1]} + R_i^{[1,0,1,1]}} + \frac{R_i^{[0,1,1,1]}}{R_i^{[0,0,0,1]} + R_i^{[0,1,1,1]}} + \frac{R_i^{[1,1,1,1]}}{R_i^{[0,0,0,1]} + R_i^{[1,1,1,1]}} \Bigg),
\end{aligned} \tag{028}
$$

$$
\begin{aligned}
\mathscr{H}\left(R_i^{[1,1,0,0]}\right) = \beta R_i^{[1,1,0,0]} & \left(\frac{S_i}{S_i + R_i^{[1,1,0,0]}} + \frac{R_i^{[1,0,0,0]}}{R_i^{[1,0,0,0]} + R_i^{[1,1,0,0]}} + \frac{R_i^{[0,1,0,0]}}{R_i^{[0,1,0,0]} + R_i^{[1,1,0,0]}}\right) + \\
& 2\frac{\beta R_i^{[1,0,0,0]} R_i^{[0,1,0,0]}}{R_i^{[1,0,0,0]} + R_i^{[0,1,0,0]}} - \beta R_i^{[1,1,0,0]} \left(\frac{R_i^{[0,0,1,0]}}{R_i^{[1,1,0,0]} + R_i^{[0,0,1,0]}} + \right. \\
& \frac{R_i^{[0,0,0,1]}}{R_i^{[1,1,0,0]} + R_i^{[0,0,0,1]}} + \frac{R_i^{[1,0,1,0]}}{R_i^{[1,1,0,0]} + R_i^{[1,0,1,0]}} + \frac{R_i^{[1,0,0,1]}}{R_i^{[1,1,0,0]} + R_i^{[1,0,0,1]}} + \\
& \frac{R_i^{[0,1,1,0]}}{R_i^{[1,1,0,0]} + R_i^{[0,1,1,0]}} + \frac{R_i^{[0,1,0,1]}}{R_i^{[1,1,0,0]} + R_i^{[0,1,0,1]}} + \frac{R_i^{[0,0,1,1]}}{R_i^{[1,1,0,0]} + R_i^{[0,0,1,1]}} + \\
& \frac{R_i^{[1,1,1,0]}}{R_i^{[1,1,0,0]} + R_i^{[1,1,1,0]}} + \frac{R_i^{[1,1,0,1]}}{R_i^{[1,1,0,0]} + R_i^{[1,1,0,1]}} + \frac{R_i^{[1,0,1,1]}}{R_i^{[1,1,0,0]} + R_i^{[1,0,1,1]}} + \\
& \left. \frac{R_i^{[0,1,1,1]}}{R_i^{[1,1,0,0]} + R_i^{[0,1,1,1]}} + \frac{R_i^{[1,1,1,1]}}{R_i^{[1,1,0,0]} + R_i^{[1,1,1,1]}}\right),
\end{aligned}
\tag{029}
$$

$$
\begin{aligned}
\mathscr{H}\left(R_i^{[1,0,1,0]}\right) = \beta R_i^{[1,0,1,0]} & \left(\frac{S_i}{S_i + R_i^{[1,0,1,0]}} + \frac{R_i^{[1,0,0,0]}}{R_i^{[1,0,0,0]} + R_i^{[1,0,1,0]}} + \frac{R_i^{[0,0,1,0]}}{R_i^{[0,0,1,0]} + R_i^{[1,0,1,0]}}\right) + \\
& 2\frac{\beta R_i^{[1,0,0,0]} R_i^{[0,0,1,0]}}{R_i^{[1,0,0,0]} + R_i^{[0,0,1,0]}} - \beta R_i^{[1,0,1,0]} \left(\frac{R_i^{[0,1,0,0]}}{R_i^{[1,0,1,0]} + R_i^{[0,1,0,0]}} + \right. \\
& \frac{R_i^{[0,0,0,1]}}{R_i^{[1,0,1,0]} + R_i^{[0,0,0,1]}} + \frac{R_i^{[1,1,0,0]}}{R_i^{[1,0,1,0]} + R_i^{[1,1,0,0]}} + \frac{R_i^{[1,0,0,1]}}{R_i^{[1,0,1,0]} + R_i^{[1,0,0,1]}} + \\
& \frac{R_i^{[0,1,1,0]}}{R_i^{[1,0,1,0]} + R_i^{[0,1,1,0]}} + \frac{R_i^{[0,1,0,1]}}{R_i^{[1,0,1,0]} + R_i^{[0,1,0,1]}} + \frac{R_i^{[0,0,1,1]}}{R_i^{[1,0,1,0]} + R_i^{[0,0,1,1]}} + \\
& \frac{R_i^{[1,1,1,0]}}{R_i^{[1,0,1,0]} + R_i^{[1,1,1,0]}} + \frac{R_i^{[1,1,0,1]}}{R_i^{[1,0,1,0]} + R_i^{[1,1,0,1]}} + \frac{R_i^{[1,0,1,1]}}{R_i^{[1,0,1,0]} + R_i^{[1,0,1,1]}} + \\
& \left. \frac{R_i^{[0,1,1,1]}}{R_i^{[1,0,1,0]} + R_i^{[0,1,1,1]}} + \frac{R_i^{[1,1,1,1]}}{R_i^{[1,0,1,0]} + R_i^{[1,1,1,1]}}\right),
\end{aligned}
\tag{030}
$$

$$
\begin{aligned}
\mathscr{H}\left(R_i^{[1,0,0,1]}\right) = \beta R_i^{[1,0,0,1]} & \left(\frac{S_i}{S_i + R_i^{[1,0,0,1]}} + \frac{R_i^{[1,0,0,0]}}{R_i^{[1,0,0,0]} + R_i^{[1,0,0,1]}} + \frac{R_i^{[0,0,0,1]}}{R_i^{[0,0,1,0]} + R_i^{[1,0,0,1]}}\right) + \\
& 2\frac{\beta R_i^{[1,0,0,0]} R_i^{[0,0,0,1]}}{R_i^{[1,0,0,0]} + R_i^{[0,0,0,1]}} - \beta R_i^{[1,0,0,1]} \left(\frac{R_i^{[0,1,0,0]}}{R_i^{[1,0,0,1]} + R_i^{[0,1,0,0]}} + \right. \\
& \frac{R_i^{[0,0,1,0]}}{R_i^{[1,0,0,1]} + R_i^{[0,0,1,0]}} + \frac{R_i^{[1,1,0,0]}}{R_i^{[1,0,0,1]} + R_i^{[1,1,0,0]}} + \frac{R_i^{[1,0,1,0]}}{R_i^{[1,0,0,1]} + R_i^{[1,0,1,0]}} + \\
& \frac{R_i^{[0,1,1,0]}}{R_i^{[1,0,0,1]} + R_i^{[0,1,1,0]}} + \frac{R_i^{[0,1,0,1]}}{R_i^{[1,0,0,1]} + R_i^{[0,1,0,1]}} + \frac{R_i^{[0,0,1,1]}}{R_i^{[1,0,0,1]} + R_i^{[0,0,1,1]}} + \\
& \frac{R_i^{[1,1,1,0]}}{R_i^{[1,0,0,1]} + R_i^{[1,1,1,0]}} + \frac{R_i^{[1,1,0,1]}}{R_i^{[1,0,0,1]} + R_i^{[1,1,0,1]}} + \frac{R_i^{[1,0,1,1]}}{R_i^{[1,0,0,1]} + R_i^{[1,0,1,1]}} + \\
& \left. \frac{R_i^{[0,1,1,1]}}{R_i^{[1,0,0,1]} + R_i^{[0,1,1,1]}} + \frac{R_i^{[1,1,1,1]}}{R_i^{[1,0,0,1]} + R_i^{[1,1,1,1]}}\right),
\end{aligned}
\tag{031}
$$

$$\mathscr{H}\left(R_i^{[0,1,1,0]}\right) = \beta R_i^{[0,1,1,0]}\left(\frac{S_i}{S_i + R_i^{[0,1,1,0]}} + \frac{R_i^{[0,1,0,0]}}{R_i^{[0,1,0,0]} + R_i^{[0,1,1,0]}} + \frac{R_i^{[0,0,1,0]}}{R_i^{[0,0,1,0]} + R_i^{[0,1,1,0]}}\right) + 2\frac{\beta R_i^{[0,1,0,0]} R_i^{[0,0,1,0]}}{R_i^{[0,1,0,0]} + R_i^{[0,0,1,0]}} - \beta R_i^{[0,1,1,0]}\left(\frac{R_i^{[1,0,0,0]}}{R_i^{[0,1,1,0]} + R_i^{[1,0,0,0]}} + \frac{R_i^{[0,0,0,1]}}{R_i^{[0,1,1,0]} + R_i^{[0,0,0,1]}} + \frac{R_i^{[1,1,0,0]}}{R_i^{[0,1,1,0]} + R_i^{[1,1,0,0]}} + \frac{R_i^{[1,0,1,0]}}{R_i^{[0,1,1,0]} + R_i^{[1,0,1,0]}} + \frac{R_i^{[1,0,0,1]}}{R_i^{[0,1,1,0]} + R_i^{[1,0,0,1]}} + \frac{R_i^{[0,1,0,1]}}{R_i^{[0,1,1,0]} + R_i^{[0,1,0,1]}} + \frac{R_i^{[0,0,1,1]}}{R_i^{[0,1,1,0]} + R_i^{[0,0,1,1]}} + \frac{R_i^{[1,1,1,0]}}{R_i^{[0,1,1,0]} + R_i^{[1,1,1,0]}} + \frac{R_i^{[1,1,0,1]}}{R_i^{[0,1,1,0]} + R_i^{[1,1,0,1]}} + \frac{R_i^{[1,0,1,1]}}{R_i^{[0,1,1,0]} + R_i^{[1,0,1,1]}} + \frac{R_i^{[0,1,1,1]}}{R_i^{[0,1,1,0]} + R_i^{[0,1,1,1]}} + \frac{R_i^{[1,1,1,1]}}{R_i^{[0,1,1,0]} + R_i^{[1,1,1,1]}}\right), \tag{032}$$

$$\mathscr{H}\left(R_i^{[0,1,0,1]}\right) = \beta R_i^{[0,1,0,1]}\left(\frac{S_i}{S_i + R_i^{[0,1,0,1]}} + \frac{R_i^{[0,1,0,0]}}{R_i^{[0,1,0,0]} + R_i^{[0,1,0,1]}} + \frac{R_i^{[0,0,0,1]}}{R_i^{[0,0,1,0]} + R_i^{[0,1,0,1]}}\right) + 2\frac{\beta R_i^{[0,1,0,0]} R_i^{[0,0,0,1]}}{R_i^{[0,1,0,0]} + R_i^{[0,0,0,1]}} - \beta R_i^{[0,1,0,1]}\left(\frac{R_i^{[1,0,0,0]}}{R_i^{[0,1,0,1]} + R_i^{[1,0,0,0]}} + \frac{R_i^{[0,0,1,0]}}{R_i^{[0,1,0,1]} + R_i^{[0,0,1,0]}} + \frac{R_i^{[1,1,0,0]}}{R_i^{[0,1,0,1]} + R_i^{[1,1,0,0]}} + \frac{R_i^{[1,0,1,0]}}{R_i^{[0,1,0,1]} + R_i^{[1,0,1,0]}} + \frac{R_i^{[1,0,0,1]}}{R_i^{[0,1,0,1]} + R_i^{[1,0,0,1]}} + \frac{R_i^{[0,1,1,0]}}{R_i^{[0,1,0,1]} + R_i^{[0,1,1,0]}} + \frac{R_i^{[0,0,1,1]}}{R_i^{[0,1,0,1]} + R_i^{[0,0,1,1]}} + \frac{R_i^{[1,1,1,0]}}{R_i^{[0,1,0,1]} + R_i^{[1,1,1,0]}} + \frac{R_i^{[1,1,0,1]}}{R_i^{[0,1,0,1]} + R_i^{[1,1,0,1]}} + \frac{R_i^{[1,0,1,1]}}{R_i^{[0,1,0,1]} + R_i^{[1,0,1,1]}} + \frac{R_i^{[0,1,1,1]}}{R_i^{[0,1,0,1]} + R_i^{[0,1,1,1]}} + \frac{R_i^{[1,1,1,1]}}{R_i^{[0,1,0,1]} + R_i^{[1,1,1,1]}}\right), \tag{033}$$

$$\mathscr{H}\left(R_i^{[0,0,1,1]}\right) = \beta R_i^{[0,0,1,1]}\left(\frac{S_i}{S_i + R_i^{[0,0,1,1]}} + \frac{R_i^{[0,0,1,0]}}{R_i^{[0,0,1,0]} + R_i^{[0,0,1,1]}} + \frac{R_i^{[0,0,0,1]}}{R_i^{[0,0,1,0]} + R_i^{[0,0,1,1]}}\right) + 2\frac{\beta R_i^{[0,0,1,0]} R_i^{[0,0,0,1]}}{R_i^{[0,0,1,0]} + R_i^{[0,0,0,1]}} - \beta R_i^{[0,0,1,1]}\left(\frac{R_i^{[1,0,0,0]}}{R_i^{[0,0,1,1]} + R_i^{[1,0,0,0]}} + \frac{R_i^{[0,1,0,0]}}{R_i^{[0,0,1,1]} + R_i^{[0,1,0,0]}} + \frac{R_i^{[1,1,0,0]}}{R_i^{[0,0,1,1]} + R_i^{[1,1,0,0]}} + \frac{R_i^{[1,0,1,0]}}{R_i^{[0,0,1,1]} + R_i^{[1,0,1,0]}} + \frac{R_i^{[1,0,0,1]}}{R_i^{[0,0,1,1]} + R_i^{[1,0,0,1]}} + \frac{R_i^{[0,1,1,0]}}{R_i^{[0,0,1,1]} + R_i^{[0,1,1,0]}} + \frac{R_i^{[0,1,0,1]}}{R_i^{[0,0,1,1]} + R_i^{[0,1,0,1]}} + \frac{R_i^{[1,1,1,0]}}{R_i^{[0,0,1,1]} + R_i^{[1,1,1,0]}} + \frac{R_i^{[1,1,0,1]}}{R_i^{[0,0,1,1]} + R_i^{[1,1,0,1]}} + \frac{R_i^{[1,0,1,1]}}{R_i^{[0,0,1,1]} + R_i^{[1,0,1,1]}} + \frac{R_i^{[0,1,1,1]}}{R_i^{[0,0,1,1]} + R_i^{[0,1,1,1]}} + \frac{R_i^{[1,1,1,1]}}{R_i^{[0,0,1,1]} + R_i^{[1,1,1,1]}}\right), \tag{034}$$

$$
\begin{aligned}
\mathscr{H}\left(R_i^{[1,1,1,0]}\right) = \beta R_i^{[1,1,1,0]} & \left(\frac{S_i}{S_i + R_i^{[1,1,1,0]}} + \frac{R_i^{[1,0,0,0]}}{R_i^{[1,0,0,0]} + R_i^{[1,1,1,0]}} + \frac{R_i^{[0,1,0,0]}}{R_i^{[0,1,0,0]} + R_i^{[1,1,1,0]}} + \right. \\
& \frac{R_i^{[0,0,1,0]}}{R_i^{[0,0,1,0]} + R_i^{[1,1,1,0]}} + \frac{R_i^{[1,1,0,0]}}{R_i^{[1,1,0,0]} + R_i^{[1,1,1,0]}} + \frac{R_i^{[1,0,1,0]}}{R_i^{[1,0,1,0]} + R_i^{[1,1,1,0]}} + \\
& \left. \frac{R_i^{[0,1,1,0]}}{R_i^{[0,1,1,0]} + R_i^{[1,1,1,0]}} \right) + \\
& \beta R_i^{[1,1,0,0]} \left(\frac{R_i^{[0,0,1,0]}}{R_i^{[0,0,1,0]} + R_i^{[1,1,0,0]}} + \frac{R_i^{[1,0,1,0]}}{R_i^{[1,0,1,0]} + R_i^{[1,1,0,0]}} + \frac{R_i^{[0,1,1,0]}}{R_i^{[1,0,1,0]} + R_i^{[1,1,0,0]}} \right) + \\
& \beta R_i^{[1,0,1,0]} \left(\frac{R_i^{[0,1,0,0]}}{R_i^{[0,1,0,0]} + R_i^{[1,0,1,0]}} + \frac{R_i^{[1,1,0,0]}}{R_i^{[1,1,0,0]} + R_i^{[1,0,1,0]}} + \frac{R_i^{[0,1,1,0]}}{R_i^{[0,1,1,0]} + R_i^{[1,0,1,0]}} \right) + \\
& \beta R_i^{[0,1,1,0]} \left(\frac{R_i^{[1,0,0,0]}}{R_i^{[1,0,0,0]} + R_i^{[0,1,1,0]}} + \frac{R_i^{[1,1,0,0]}}{R_i^{[1,1,0,0]} + R_i^{[0,1,1,0]}} + \frac{R_i^{[1,0,1,0]}}{R_i^{[1,0,1,0]} + R_i^{[0,1,1,0]}} \right) + \\
& \frac{\beta R_i^{[1,0,0,0]} R_i^{[0,1,1,0]}}{R_i^{[1,0,0,0]} + R_i^{[0,1,1,0]}} + \frac{\beta R_i^{[0,1,0,0]} R_i^{[1,0,1,0]}}{R_i^{[0,1,0,0]} + R_i^{[1,0,1,0]}} + \frac{\beta R_i^{[0,0,1,0]} R_i^{[1,1,0,0]}}{R_i^{[0,0,1,0]} + R_i^{[1,1,0,0]}} - \\
& \beta R_i^{[1,1,1,0]} \left(\frac{R_i^{[0,0,0,1]}}{R_i^{[0,0,0,1]} + R_i^{[1,1,1,0]}} + \frac{R_i^{[1,0,0,1]}}{R_i^{[1,0,0,1]} + R_i^{[1,1,1,0]}} + \frac{R_i^{[0,1,0,1]}}{R_i^{[0,1,0,1]} + R_i^{[1,1,1,0]}} + \right. \\
& \frac{R_i^{[0,0,1,1]}}{R_i^{[0,0,1,1]} + R_i^{[1,1,1,0]}} + \frac{R_i^{[1,1,0,1]}}{R_i^{[1,1,0,1]} + R_i^{[1,1,1,0]}} + \frac{R_i^{[1,0,1,1]}}{R_i^{[1,0,1,1]} + R_i^{[1,1,1,0]}} + \\
& \left. \frac{R_i^{[0,1,1,1]}}{R_i^{[0,1,1,1]} + R_i^{[1,1,1,0]}} + \frac{R_i^{[1,1,1,1]}}{R_i^{[1,1,1,1]} + R_i^{[1,1,1,0]}} \right),
\end{aligned}
\tag{035}
$$

$$
\begin{aligned}
\mathscr{H}\left(R_i^{[1,1,0,1]}\right) = {} & \beta R_i^{[1,1,0,1]} \left(\frac{S_i}{S_i + R_i^{[1,1,0,1]}} + \frac{R_i^{[1,0,0,0]}}{R_i^{[1,0,0,0]} + R_i^{[1,1,0,1]}} + \frac{R_i^{[0,1,0,0]}}{R_i^{[0,1,0,0]} + R_i^{[1,1,0,1]}} + \right. \\
& \frac{R_i^{[0,0,0,1]}}{R_i^{[0,0,0,1]} + R_i^{[1,1,0,1]}} + \frac{R_i^{[1,1,0,0]}}{R_i^{[1,1,0,0]} + R_i^{[1,1,0,1]}} + \frac{R_i^{[1,0,0,1]}}{R_i^{[1,0,0,1]} + R_i^{[1,1,0,1]}} + \\
& \left. \frac{R_i^{[0,1,0,1]}}{R_i^{[0,1,0,1]} + R_i^{[1,1,0,1]}} \right) + \\
& \beta R_i^{[1,1,0,0]} \left(\frac{R_i^{[0,0,0,1]}}{R_i^{[0,0,0,1]} + R_i^{[1,1,0,0]}} + \frac{R_i^{[1,0,0,1]}}{R_i^{[1,0,0,1]} + R_i^{[1,1,0,0]}} + \frac{R_i^{[0,1,0,1]}}{R_i^{[0,1,0,1]} + R_i^{[1,1,0,0]}} \right) + \\
& \beta R_i^{[1,0,0,1]} \left(\frac{R_i^{[0,1,0,0]}}{R_i^{[0,1,0,0]} + R_i^{[1,0,0,1]}} + \frac{R_i^{[1,1,0,0]}}{R_i^{[1,1,0,0]} + R_i^{[1,0,0,1]}} + \frac{R_i^{[0,1,0,1]}}{R_i^{[0,1,0,1]} + R_i^{[1,0,0,1]}} \right) + \\
& \beta R_i^{[0,1,0,1]} \left(\frac{R_i^{[1,0,0,0]}}{R_i^{[1,0,0,0]} + R_i^{[0,1,0,1]}} + \frac{R_i^{[1,1,0,0]}}{R_i^{[1,1,0,0]} + R_i^{[0,1,0,1]}} + \frac{R_i^{[1,0,0,1]}}{R_i^{[1,0,0,1]} + R_i^{[0,1,0,1]}} \right) + \\
& \frac{\beta R_i^{[1,0,0,0]} R_i^{[0,1,0,1]}}{R_i^{[1,0,0,0]} + R_i^{[0,1,0,1]}} + \frac{\beta R_i^{[0,1,0,0]} R_i^{[1,0,0,1]}}{R_i^{[0,1,0,0]} + R_i^{[1,0,0,1]}} + \frac{\beta R_i^{[0,0,0,1]} R_i^{[1,1,0,0]}}{R_i^{[0,0,0,1]} + R_i^{[1,1,0,0]}} - \\
& \beta R_i^{[1,1,0,1]} \left(\frac{R_i^{[0,0,1,0]}}{R_i^{[0,0,1,0]} + R_i^{[1,1,0,1]}} + \frac{R_i^{[1,0,1,0]}}{R_i^{[1,0,1,0]} + R_i^{[1,1,0,1]}} + \frac{R_i^{[0,1,1,0]}}{R_i^{[0,1,1,0]} + R_i^{[1,1,0,1]}} + \right. \\
& \frac{R_i^{[0,0,1,1]}}{R_i^{[0,0,1,1]} + R_i^{[1,1,0,1]}} + \frac{R_i^{[1,1,1,0]}}{R_i^{[1,1,1,0]} + R_i^{[1,1,0,1]}} + \frac{R_i^{[1,0,1,1]}}{R_i^{[1,0,1,1]} + R_i^{[1,1,0,1]}} + \\
& \left. \frac{R_i^{[0,1,1,1]}}{R_i^{[0,1,1,1]} + R_i^{[1,1,0,1]}} + \frac{R_i^{[1,1,1,1]}}{R_i^{[1,1,1,1]} + R_i^{[1,1,0,1]}} \right),
\end{aligned}
\tag{036}
$$

$$
\begin{aligned}
\mathscr{H}\left(R_i^{[1,0,1,1]}\right) = \beta R_i^{[1,0,1,1]} & \left(\frac{S_i}{S_i + R_i^{[1,0,1,1]}} + \frac{R_i^{[1,0,0,0]}}{R_i^{[1,0,0,0]} + R_i^{[1,0,1,1]}} + \frac{R_i^{[0,0,1,0]}}{R_i^{[0,0,1,0]} + R_i^{[1,0,1,1]}} + \right. \\
& \frac{R_i^{[0,0,0,1]}}{R_i^{[0,0,0,1]} + R_i^{[1,0,1,1]}} + \frac{R_i^{[1,0,1,0]}}{R_i^{[1,0,1,0]} + R_i^{[1,0,1,1]}} + \frac{R_i^{[1,0,0,1]}}{R_i^{[1,0,0,1]} + R_i^{[1,0,1,1]}} + \\
& \left. \frac{R_i^{[0,0,1,1]}}{R_i^{[0,0,1,1]} + R_i^{[1,0,1,1]}} \right) + \\
& \beta R_i^{[1,0,1,0]} \left(\frac{R_i^{[0,0,0,1]}}{R_i^{[0,0,0,1]} + R_i^{[1,0,1,0]}} + \frac{R_i^{[1,0,0,1]}}{R_i^{[1,0,0,1]} + R_i^{[1,0,1,0]}} + \frac{R_i^{[0,0,1,1]}}{R_i^{[0,0,1,1]} + R_i^{[1,0,1,0]}} \right) + \\
& \beta R_i^{[1,0,0,1]} \left(\frac{R_i^{[0,0,1,0]}}{R_i^{[0,0,1,0]} + R_i^{[1,0,0,1]}} + \frac{R_i^{[1,0,1,0]}}{R_i^{[1,0,1,0]} + R_i^{[1,0,0,1]}} + \frac{R_i^{[0,0,1,1]}}{R_i^{[0,0,1,1]} + R_i^{[1,0,0,1]}} \right) + \\
& \beta R_i^{[0,0,1,1]} \left(\frac{R_i^{[1,0,0,0]}}{R_i^{[1,0,0,0]} + R_i^{[0,0,1,1]}} + \frac{R_i^{[1,0,1,0]}}{R_i^{[1,0,1,0]} + R_i^{[0,0,1,1]}} + \frac{R_i^{[1,0,0,1]}}{R_i^{[1,0,0,1]} + R_i^{[0,0,1,1]}} \right) + \\
& \frac{\beta R_i^{[1,0,0,0]} R_i^{[0,0,1,1]}}{R_i^{[1,0,0,0]} + R_i^{[0,0,1,1]}} + \frac{\beta R_i^{[0,0,1,0]} R_i^{[1,0,0,1]}}{R_i^{[0,0,1,0]} + R_i^{[1,0,0,1]}} + \frac{\beta R_i^{[0,0,0,1]} R_i^{[1,0,1,0]}}{R_i^{[0,0,0,1]} + R_i^{[1,0,1,0]}} - \\
& \beta R_i^{[1,0,1,1]} \left(\frac{R_i^{[0,1,0,0]}}{R_i^{[0,1,0,0]} + R_i^{[1,0,1,1]}} + \frac{R_i^{[1,1,0,0]}}{R_i^{[1,1,0,0]} + R_i^{[1,0,1,1]}} + \frac{R_i^{[0,1,1,0]}}{R_i^{[0,1,1,0]} + R_i^{[1,0,1,1]}} + \right. \\
& \frac{R_i^{[0,1,0,1]}}{R_i^{[0,1,0,1]} + R_i^{[1,0,1,1]}} + \frac{R_i^{[1,1,1,0]}}{R_i^{[1,1,1,0]} + R_i^{[1,0,1,1]}} + \frac{R_i^{[1,1,0,1]}}{R_i^{[1,1,0,1]} + R_i^{[1,0,1,1]}} + \\
& \left. \frac{R_i^{[0,1,1,1]}}{R_i^{[0,1,1,1]} + R_i^{[1,0,1,1]}} + \frac{R_i^{[1,1,1,1]}}{R_i^{[1,1,1,1]} + R_i^{[1,0,1,1]}} \right),
\end{aligned}
\tag{037}
$$

$$
\begin{aligned}
\mathscr{H}\left(R_i^{[0,1,1,1]}\right) = \beta R_i^{[0,1,1,1]} & \left(\frac{S_i}{S_i + R_i^{[0,1,1,1]}} + \frac{R_i^{[0,1,0,0]}}{R_i^{[0,1,0,0]} + R_i^{[0,1,1,1]}} + \frac{R_i^{[0,0,1,0]}}{R_i^{[0,0,1,0]} + R_i^{[0,1,1,1]}} + \right. \\
& \frac{R_i^{[0,0,0,1]}}{R_i^{[0,0,0,1]} + R_i^{[0,1,1,1]}} + \frac{R_i^{[0,1,1,0]}}{R_i^{[0,1,1,0]} + R_i^{[0,1,1,1]}} + \frac{R_i^{[0,1,0,1]}}{R_i^{[0,1,0,1]} + R_i^{[0,1,1,1]}} + \\
& \left. \frac{R_i^{[0,0,1,1]}}{R_i^{[0,0,1,1]} + R_i^{[0,1,1,1]}} \right) + \\
& \beta R_i^{[0,1,1,0]} \left(\frac{R_i^{[0,0,0,1]}}{R_i^{[0,0,0,1]} + R_i^{[0,1,1,0]}} + \frac{R_i^{[0,1,0,1]}}{R_i^{[0,1,0,1]} + R_i^{[0,1,1,0]}} + \frac{R_i^{[0,0,1,1]}}{R_i^{[0,0,1,1]} + R_i^{[0,1,1,0]}} \right) + \\
& \beta R_i^{[0,1,0,1]} \left(\frac{R_i^{[0,0,1,0]}}{R_i^{[0,0,1,0]} + R_i^{[0,1,0,1]}} + \frac{R_i^{[0,1,1,0]}}{R_i^{[0,1,1,0]} + R_i^{[0,1,0,1]}} + \frac{R_i^{[0,0,1,1]}}{R_i^{[0,0,1,1]} + R_i^{[0,1,0,1]}} \right) + \\
& \beta R_i^{[0,0,1,1]} \left(\frac{R_i^{[0,1,0,0]}}{R_i^{[0,1,0,0]} + R_i^{[0,0,1,1]}} + \frac{R_i^{[0,1,1,0]}}{R_i^{[0,1,1,0]} + R_i^{[0,0,1,1]}} + \frac{R_i^{[0,1,0,1]}}{R_i^{[0,1,0,1]} + R_i^{[0,0,1,1]}} \right) + \\
& \frac{\beta R_i^{[0,1,0,0]} R_i^{[0,0,1,1]}}{R_i^{[0,1,0,0]} + R_i^{[0,0,1,1]}} + \frac{\beta R_i^{[0,0,1,0]} R_i^{[0,1,0,1]}}{R_i^{[0,0,1,0]} + R_i^{[0,1,0,1]}} + \frac{\beta R_i^{[0,0,0,1]} R_i^{[0,1,1,0]}}{R_i^{[0,0,0,1]} + R_i^{[0,1,1,0]}} - \\
& \beta R_i^{[0,1,1,1]} \left(\frac{R_i^{[1,0,0,0]}}{R_i^{[1,0,0,0]} + R_i^{[0,1,1,1]}} + \frac{R_i^{[1,1,0,0]}}{R_i^{[1,1,0,0]} + R_i^{[0,1,1,1]}} + \frac{R_i^{[1,0,1,0]}}{R_i^{[1,0,1,0]} + R_i^{[0,1,1,1]}} + \right. \\
& \frac{R_i^{[1,0,0,1]}}{R_i^{[1,0,0,1]} + R_i^{[0,1,1,1]}} + \frac{R_i^{[1,1,1,0]}}{R_i^{[1,1,1,0]} + R_i^{[0,1,1,1]}} + \frac{R_i^{[1,1,0,1]}}{R_i^{[1,1,0,1]} + R_i^{[0,1,1,1]}} + \\
& \left. \frac{R_i^{[1,0,1,1]}}{R_i^{[1,0,1,1]} + R_i^{[0,1,1,1]}} + \frac{R_i^{[1,1,1,1]}}{R_i^{[1,1,1,1]} + R_i^{[0,1,1,1]}} \right),
\end{aligned}
\tag{038}
$$

$$
\begin{aligned}
\mathscr{H}\left(R_i^{[1,1,1,1]}\right) = {} & \frac{\beta S_i R_i^{[1,1,1,1]}}{S_i + R_i^{[1,1,1,1]}} + \beta R_i^{[1,0,0,0]} \Bigg(\frac{R_i^{[1,1,1,1]}}{R_i^{[1,0,0,0]} + R_i^{[1,1,1,1]}} + \frac{R_i^{[0,1,1,1]}}{R_i^{[1,0,0,0]} + R_i^{[0,1,1,1]}} \Bigg) + \beta R_i^{[0,1,0,0]} \Bigg(\frac{R_i^{[1,1,1,1]}}{R_i^{[0,1,0,0]} + R_i^{[1,1,1,1]}} + \\
& \frac{R_i^{[1,0,1,1]}}{R_i^{[0,1,0,0]} + R_i^{[1,0,1,1]}} \Bigg) + \beta R_i^{[0,0,1,0]} \Bigg(\frac{R_i^{[1,1,1,1]}}{R_i^{[0,0,1,0]} + R_i^{[1,1,1,1]}} + \frac{R_i^{[1,1,0,1]}}{R_i^{[0,0,1,0]} + R_i^{[1,1,0,1]}} \Bigg) + \\
& \beta R_i^{[0,0,0,1]} \Bigg(\frac{R_i^{[1,1,1,1]}}{R_i^{[0,0,0,1]} + R_i^{[1,1,1,1]}} + \frac{R_i^{[1,1,1,0]}}{R_i^{[0,0,0,1]} + R_i^{[1,1,1,0]}} \Bigg) + \beta R_i^{[1,1,0,0]} \Bigg(\frac{R_i^{[1,1,1,1]}}{R_i^{[1,1,0,0]} + R_i^{[1,1,1,1]}} + \\
& \frac{R_i^{[1,0,1,1]}}{R_i^{[1,1,0,0]} + R_i^{[1,0,1,1]}} + \frac{R_i^{[0,1,1,1]}}{R_i^{[1,1,0,0]} + R_i^{[0,1,1,1]}} + \frac{R_i^{[0,0,1,1]}}{R_i^{[1,1,0,0]} + R_i^{[0,0,1,1]}} \Bigg) + \beta R_i^{[1,0,1,0]} \Bigg(\frac{R_i^{[1,1,1,1]}}{R_i^{[1,0,1,0]} + R_i^{[1,1,1,1]}} + \\
& \frac{R_i^{[1,1,0,1]}}{R_i^{[1,0,1,0]} + R_i^{[1,1,0,1]}} + \frac{R_i^{[0,1,1,1]}}{R_i^{[1,0,1,0]} + R_i^{[0,1,1,1]}} + \frac{R_i^{[0,1,0,1]}}{R_i^{[1,0,1,0]} + R_i^{[0,1,0,1]}} \Bigg) + \beta R_i^{[1,0,0,1]} \Bigg(\frac{R_i^{[1,1,1,1]}}{R_i^{[1,0,0,1]} + R_i^{[1,1,1,1]}} + \\
& \frac{R_i^{[1,1,1,0]}}{R_i^{[1,0,0,1]} + R_i^{[1,1,1,0]}} + \frac{R_i^{[0,1,1,1]}}{R_i^{[1,0,0,1]} + R_i^{[0,1,1,1]}} + \frac{R_i^{[0,1,1,0]}}{R_i^{[1,0,0,1]} + R_i^{[0,1,1,0]}} \Bigg) + \beta R_i^{[0,1,1,0]} \Bigg(\frac{R_i^{[1,1,1,1]}}{R_i^{[0,1,1,0]} + R_i^{[1,1,1,1]}} + \\
& \frac{R_i^{[1,1,0,1]}}{R_i^{[0,1,1,0]} + R_i^{[1,1,0,1]}} + \frac{R_i^{[1,0,1,1]}}{R_i^{[0,1,1,0]} + R_i^{[1,0,1,1]}} + \frac{R_i^{[1,00,1]}}{R_i^{[0,1,1,0]} + R_i^{[1,0,0,1]}} \Bigg) + \beta R_i^{[0,1,0,1]} \Bigg(\frac{R_i^{[1,1,1,1]}}{R_i^{[0,1,0,1]} + R_i^{[1,1,1,1]}} + \\
& \frac{R_i^{[1,1,1,0]}}{R_i^{[0,1,0,1]} + R_i^{[1,1,1,0]}} + \frac{R_i^{[1,0,1,1]}}{R_i^{[0,1,0,1]} + R_i^{[1,0,1,1]}} + \frac{R_i^{[1,0,1,0]}}{R_i^{[0,1,0,1]} + R_i^{[1,0,1,0]}} \Bigg) + \beta R_i^{[0,0,1,1]} \Bigg(\frac{R_i^{[1,1,1,1]}}{R_i^{[0,0,1,1]} + R_i^{[1,1,1,1]}} + \\
& \frac{R_i^{[1,1,1,0]}}{R_i^{[0,0,1,1]} + R_i^{[1,1,1,0]}} + \frac{R_i^{[1,1,0,1]}}{R_i^{[0,0,1,1]} + R_i^{[1,1,0,1]}} + \frac{R_i^{[1,1,0,0]}}{R_i^{[0,0,1,1]} + R_i^{[1,1,0,0]}} \Bigg) + \beta R_i^{[1,1,1,0]} \Bigg(\frac{R_i^{[1,1,1,1]}}{R_i^{[1,1,1,0]} + R_i^{[1,1,1,1]}} + \\
& \frac{R_i^{[1,1,0,1]}}{R_i^{[1,1,1,0]} + R_i^{[1,1,0,1]}} + \frac{R_i^{[1,0,1,1]}}{R_i^{[1,1,1,0]} + R_i^{[1,0,1,1]}} + \frac{R_i^{[0,1,1,1]}}{R_i^{[1,1,1,0]} + R_i^{[0,1,1,1]}} + \frac{R_i^{[1,0,0,1]}}{R_i^{[1,1,1,0]} + R_i^{[1,0,0,1]}} + \\
& \frac{R_i^{[0,1,0,1]}}{R_i^{[1,1,1,0]} + R_i^{[0,1,0,1]}} + \frac{R_i^{[0,0,1,1]}}{R_i^{[1,1,1,0]} + R_i^{[0,0,1,1]}} + \frac{R_i^{[0,0,0,1]}}{R_i^{[1,1,1,0]} + R_i^{[0,0,0,1]}} \Bigg) + \beta R_i^{[1,1,0,1]} \Bigg(\frac{R_i^{[1,1,1,1]}}{R_i^{[1,1,0,1]} + R_i^{[1,1,1,1]}} + \\
& \frac{R_i^{[1,1,1,0]}}{R_i^{[1,1,0,1]} + R_i^{[1,1,1,0]}} + \frac{R_i^{[1,0,1,1]}}{R_i^{[1,1,0,1]} + R_i^{[1,0,1,1]}} + \frac{R_i^{[0,1,1,1]}}{R_i^{[1,1,0,1]} + R_i^{[0,1,1,1]}} + \frac{R_i^{[1,0,1,0]}}{R_i^{[1,1,0,1]} + R_i^{[1,0,1,0]}} + \\
& \frac{R_i^{[0,1,1,0]}}{R_i^{[1,1,0,1]} + R_i^{[0,1,1,0]}} + \frac{R_i^{[0,0,1,1]}}{R_i^{[1,1,0,1]} + R_i^{[0,0,1,1]}} + \frac{R_i^{[0,0,1,0]}}{R_i^{[1,1,0,1]} + R_i^{[0,0,1,0]}} \Bigg) + \beta R_i^{[1,0,1,1]} \Bigg(\frac{R_i^{[1,1,1,1]}}{R_i^{[1,0,1,1]} + R_i^{[1,1,1,1]}} + \\
& \frac{R_i^{[1,1,1,0]}}{R_i^{[1,0,1,1]} + R_i^{[1,1,1,0]}} + \frac{R_i^{[1,1,0,1]}}{R_i^{[1,0,1,1]} + R_i^{[1,1,0,1]}} + \frac{R_i^{[0,1,1,1]}}{R_i^{[1,0,1,1]} + R_i^{[0,1,1,1]}} + \frac{R_i^{[1,1,0,0]}}{R_i^{[1,0,1,1]} + R_i^{[1,1,0,0]}} + \\
& \frac{R_i^{[0,1,1,0]}}{R_i^{[1,0,1,1]} + R_i^{[0,1,1,0]}} + \frac{R_i^{[0,1,0,1]}}{R_i^{[1,0,1,1]} + R_i^{[0,1,0,1]}} + \frac{R_i^{[0,1,0,0]}}{R_i^{[1,0,1,1]} + R_i^{[0,1,0,0]}} \Bigg) + \beta R_i^{[0,1,1,1]} \Bigg(\frac{R_i^{[1,1,1,1]}}{R_i^{[0,1,1,1]} + R_i^{[1,1,1,1]}} + \\
& \frac{R_i^{[1,1,1,0]}}{R_i^{[0,1,1,1]} + R_i^{[1,1,1,0]}} + \frac{R_i^{[1,1,0,1]}}{R_i^{[0,1,1,1]} + R_i^{[1,1,0,1]}} + \frac{R_i^{[1,0,1,1]}}{R_i^{[0,1,1,1]} + R_i^{[1,0,1,1]}} + \frac{R_i^{[1,1,0,0]}}{R_i^{[0,1,1,1]} + R_i^{[1,1,0,0]}} + \\
& \frac{R_i^{[1,0,1,0]}}{R_i^{[0,1,1,1]} + R_i^{[1,0,1,0]}} + \frac{R_i^{[1,0,0,1]}}{R_i^{[0,1,1,1]} + R_i^{[1,0,0,1]}} + \frac{R_i^{[1,0,0,0]}}{R_i^{[0,1,1,1]} + R_i^{[1,0,0,0]}} \Bigg).
\end{aligned}
\tag{039}
$$

The dynamics of each bacterial population in the farm flow system can then be described by the system of equations defined by:

$$\frac{dR_i^{[x_1,x_2,x_3,x_4]}}{dt} = \mathscr{F}(R_i^{[x_1,x_2,x_3,x_4]}) + \mathscr{D}(R_i^{[x_1,x_2,x_3,x_4]}) + \mathscr{G}(R_i^{[x_1,x_2,x_3,x_4]}) + \mathscr{H}(R_i^{[x_1,x_2,x_3,x_4]}), \quad \text{where } i \in \{\text{dairy, heifer, UR, muck, eff., tank}\} \;\&\; [x_1, x_2, x_3, x_4] \in \{0,1\}^4. \tag{040}$$

Supplementary Text 2: Model Equations for Chromosomal Resistance

In the case where cefelexin-resistance is chromosomally encoded, the volume, metal and antibiotic model equations remain the same ((01)-(018)), however, the model equations describing the bacterial dynamics are different: since they are now located on the chromosome, cefelexin-resistance genes no longer bear a fitness cost (i.e. $\alpha_{[\text{Cex.}]} = 0$) and also cannot be passed on to other cells via HGT. The function $\mathscr{H}^{[0,0,0,1]} : \Omega_i \to \mathbb{R}$ describes the horizontal gene transfer processes in this case and is defined by the set of equations (01)-(016).

$$
\begin{aligned}
\mathscr{H}^{[0,0,0,1]}\left(S_i\right) = -\beta S_i \Bigg(& \frac{R_i^{[1,0,0,0]}}{S_i + R_i^{[1,0,0,0]}} + \frac{R_i^{[0,1,0,0]}}{S_i + R_i^{[0,1,0,0]}} + \frac{R_i^{[0,0,1,0]}}{S_i + R_i^{[0,0,1,0]}} + \frac{R_i^{[1,1,0,0]}}{S_i + R_i^{[1,1,0,0]}} + \\
& \frac{R_i^{[1,0,1,0]}}{S_i + R_i^{[1,0,1,0]}} + \frac{R_i^{[1,0,0,1]}}{S_i + R_i^{[1,0,0,1]}} + \frac{R_i^{[0,1,1,0]}}{S_i + R_i^{[0,1,1,0]}} + \frac{R_i^{[0,1,0,1]}}{S_i + R_i^{[0,1,0,1]}} + \\
& \frac{R_i^{[0,0,1,1]}}{S_i + R_i^{[0,0,1,1]}} + \frac{R_i^{[1,1,1,0]}}{S_i + R_i^{[1,1,1,0]}} + \frac{R_i^{[1,1,0,1]}}{S_i + R_i^{[1,1,0,1]}} + \frac{R_i^{[1,0,1,1]}}{S_i + R_i^{[1,0,1,1]}} + \\
& \frac{R_i^{[0,1,1,1]}}{S_i + R_i^{[0,1,1,1]}} + \frac{R_i^{[1,1,1,1]}}{S_i + R_i^{[1,1,1,1]}} \Bigg),
\end{aligned}
\tag{01}
$$

$$
\begin{aligned}
\mathscr{H}^{[0,0,0,1]}\left(R_i^{[1,0,0,0]}\right) = & \frac{\beta S_i R_i^{[1,0,0,0]}}{S_i + R_i^{[1,0,0,0]}} + \frac{\beta S_i R_i^{[1,0,0,1]}}{S_i + R_i^{[1,0,0,1]}} - \beta R_i^{[1,0,0,0]} \Bigg(\frac{R_i^{[0,1,0,0]}}{R_i^{[1,0,0,0]} + R_i^{[0,1,0,0]}} + \\
& \frac{R_i^{[0,0,1,0]}}{R_i^{[1,0,0,0]} + R_i^{[0,0,1,0]}} + \frac{R_i^{[1,1,0,0]}}{R_i^{[1,0,0,0]} + R_i^{[1,1,0,0]}} + \frac{R_i^{[1,0,1,0]}}{R_i^{[1,0,0,0]} + R_i^{[1,0,1,0]}} + \\
& \frac{R_i^{[0,1,1,0]}}{R_i^{[1,0,0,0]} + R_i^{[0,1,1,0]}} + \frac{R_i^{[0,1,0,1]}}{R_i^{[1,0,0,0]} + R_i^{[0,1,0,1]}} + \frac{R_i^{[0,0,1,1]}}{R_i^{[1,0,0,0]} + R_i^{[0,0,1,1]}} + \\
& \frac{R_i^{[1,1,1,0]}}{R_i^{[1,0,0,0]} + R_i^{[1,1,1,0]}} + \frac{R_i^{[1,1,0,1]}}{R_i^{[1,0,0,0]} + R_i^{[1,1,0,1]}} + \frac{R_i^{[1,0,1,1]}}{R_i^{[1,0,0,0]} + R_i^{[1,0,1,1]}} + \\
& \frac{R_i^{[0,1,1,1]}}{R_i^{[1,0,0,0]} + R_i^{[0,1,1,1]}} + \frac{R_i^{[1,1,1,1]}}{R_i^{[1,0,0,0]} + R_i^{[1,1,1,1]}} \Bigg),
\end{aligned}
\tag{02}
$$

$$
\begin{aligned}
\mathscr{H}^{[0,0,0,1]}\left(R_i^{[0,1,0,0]}\right) = & \frac{\beta S_i R_i^{[0,1,0,0]}}{S_i + R_i^{[0,1,0,0]}} + \frac{\beta S_i R_i^{[0,1,0,1]}}{S_i + R_i^{[0,1,0,1]}} - \beta R_i^{[0,1,0,0]} \Bigg(\frac{R_i^{[1,0,0,0]}}{R_i^{[1,0,0,0]} + R_i^{[0,1,0,0]}} + \\
& \frac{R_i^{[0,0,1,0]}}{R_i^{[0,1,0,0]} + R_i^{[0,0,1,0]}} + \frac{R_i^{[1,1,0,0]}}{R_i^{[0,1,0,0]} + R_i^{[1,1,0,0]}} + \frac{R_i^{[1,0,1,0]}}{R_i^{[0,1,0,0]} + R_i^{[1,0,1,0]}} + \\
& \frac{R_i^{[1,0,0,1]}}{R_i^{[0,1,0,0]} + R^{[1,0,0,1]}} + \frac{R_i^{[0,1,1,0]}}{R_i^{[0,1,0,0]} + R_i^{[0,1,1,0]}} + \frac{R_i^{[0,0,1,1]}}{R_i^{[0,1,0,0]} + R_i^{[0,0,1,1]}} + \\
& \frac{R_i^{[1,1,1,0]}}{R_i^{[0,1,0,0]} + R_i^{[1,1,1,0]}} + \frac{R_i^{[1,1,0,1]}}{R_i^{[0,1,0,0]} + R_i^{[1,1,0,1]}} + \frac{R_i^{[1,0,1,1]}}{R_i^{[0,1,0,0]} + R_i^{[1,0,1,1]}} + \\
& \frac{R_i^{[0,1,1,1]}}{R_i^{[0,1,0,0]} + R_i^{[0,1,1,1]}} + \frac{R_i^{[1,1,1,1]}}{R_i^{[0,1,0,0]} + R_i^{[1,1,1,1]}} \Bigg),
\end{aligned}
\tag{03}
$$

$$
\begin{aligned}
\mathscr{H}^{[0,0,0,1]}\left(R_i^{[0,0,1,0]}\right) = {} & \frac{\beta S_i R_i^{[0,0,1,0]}}{S_i + R_i^{[0,0,1,0]}} + \frac{\beta S_i R_i^{[0,0,1,1]}}{S_i + R_i^{[0,0,1,1]}} - \beta R_i^{[0,0,1,0]} \left(\frac{R_i^{[1,0,0,0]}}{R_i^{[1,0,0,0]} + R_i^{[0,0,1,0]}} + \right. \\
& \frac{R_i^{[0,1,0,0]}}{R_i^{[0,1,0,0]} + R_i^{[0,0,1,0]}} + \frac{R_i^{[1,1,0,0]}}{R_i^{[0,0,1,0]} + R_i^{[1,1,0,0]}} + \frac{R_i^{[1,0,1,0]}}{R_i^{[0,0,1,0]} + R_i^{[1,0,1,0]}} + \\
& \frac{R_i^{[1,0,0,1]}}{R_i^{[0,0,1,0]} + R_i^{[1,0,0,1]}} + \frac{R_i^{[0,1,1,0]}}{R_i^{[0,0,1,0]} + R_i^{[0,1,1,0]}} + \frac{R_i^{[0,1,0,1]}}{R_i^{[0,0,1,0]} + R_i^{[0,1,0,1]}} + \\
& \frac{R_i^{[1,1,1,0]}}{R_i^{[0,0,1,0]} + R_i^{[1,1,1,0]}} + \frac{R_i^{[1,1,0,1]}}{R_i^{[0,0,1,0]} + R_i^{[1,1,0,1]}} + \frac{R_i^{[1,0,1,1]}}{R_i^{[0,0,1,0]} + R_i^{[1,0,1,1]}} + \\
& \left. \frac{R_i^{[0,1,1,1]}}{R_i^{[0,0,1,0]} + R_i^{[0,1,1,1]}} + \frac{R_i^{[1,1,1,1]}}{R_i^{[0,0,1,0]} + R_i^{[1,1,1,1]}} \right),
\end{aligned} \tag{04}
$$

$$
\begin{aligned}
\mathscr{H}^{[0,0,0,1]}\left(R_i^{[0,0,0,1]}\right) = {} & -\beta R_i^{[0,0,0,1]} \left(\frac{R_i^{[1,0,0,0]}}{R_i^{[0,0,0,1]} + R_i^{[1,0,0,0]}} + \frac{R_i^{[0,1,0,0]}}{R_i^{[0,0,0,1]} + R_i^{[0,1,0,0]}} + \right. \\
& \frac{R_i^{[0,0,1,0]}}{R_i^{[0,0,0,1]} + R_i^{[0,0,1,0]}} + \frac{R_i^{[1,1,0,0]}}{R_i^{[0,0,0,1]} + R_i^{[1,1,0,0]}} + \frac{R_i^{[1,0,1,0]}}{R_i^{[0,0,0,1]} + R_i^{[1,0,1,0]}} + \\
& \frac{R_i^{[1,0,0,1]}}{R_i^{[0,0,0,1]} + R_i^{[1,0,0,1]}} + \frac{R_i^{[0,1,1,0]}}{R_i^{[0,0,0,1]} + R_i^{[0,1,1,0]}} + \frac{R_i^{[0,1,0,1]}}{R_i^{[0,0,0,1]} + R_i^{[0,1,0,1]}} + \\
& \frac{R_i^{[0,0,1,1]}}{R_i^{[0,0,0,1]} + R_i^{[0,0,1,1]}} + \frac{R_i^{[1,1,1,0]}}{R_i^{[0,0,0,1]} + R_i^{[1,1,1,0]}} + \frac{R_i^{[1,1,0,1]}}{R_i^{[0,0,0,1]} + R_i^{[1,1,0,1]}} + \\
& \left. \frac{R_i^{[1,0,1,1]}}{R_i^{[0,0,0,1]} + R_i^{[1,0,1,1]}} + \frac{R_i^{[0,1,1,1]}}{R_i^{[0,0,0,1]} + R_i^{[0,1,1,1]}} + \frac{R_i^{[1,1,1,1]}}{R_i^{[0,0,0,1]} + R_i^{[1,1,1,1]}} \right),
\end{aligned} \tag{05}
$$

$$
\begin{aligned}
\mathscr{H}^{[0,0,0,1]}\left(R_i^{[1,1,0,0]}\right) = {} & \frac{\beta S_i R_i^{[1,1,0,0]}}{S_i + R_i^{[1,1,0,0]}} + \frac{\beta S_i R_i^{[1,1,0,1]}}{S_i + R_i^{[1,1,0,1]}} + 2\frac{\beta R^{[1,0,0,0]} R_i^{[0,1,0,0]}}{R^{[1,0,0,0]} + R_i^{[0,1,0,0]}} + \\
& \frac{\beta R^{[1,0,0,0]} R_i^{[1,1,0,0]}}{R^{[1,0,0,0]} + R_i^{[1,1,0,0]}} + \frac{\beta R^{[0,1,0,0]} R_i^{[1,1,0,0]}}{R^{[0,1,0,0]} + R_i^{[1,1,0,0]}} + \frac{\beta R^{[1,0,0,0]} R_i^{[0,1,0,1]}}{R^{[1,0,0,0]} + R_i^{[0,1,0,1]}} + \\
& \frac{\beta R^{[0,1,0,0]} R_i^{[1,0,0,1]}}{R^{[0,1,0,0]} + R_i^{[1,0,0,1]}} + \frac{\beta R^{[1,0,0,0]} R_i^{[1,1,0,1]}}{R^{[1,0,0,0]} + R_i^{[1,1,0,1]}} + \frac{\beta R^{[0,1,0,0]} R_i^{[1,1,0,1]}}{R^{[0,1,0,0]} + R_i^{[1,1,0,1]}} - \\
& \beta R_i^{[1,1,0,0]} \left(\frac{R_i^{[0,0,1,0]}}{R_i^{[0,0,1,0]} + R^{[1,1,0,0]}} + \frac{R_i^{[1,0,1,0]}}{R_i^{[1,1,0,0]} + R_i^{[1,0,1,0]}} + \right. \\
& \frac{R_i^{[0,1,1,0]}}{R_i^{[1,1,0,0]} + R_i^{[0,1,1,0]}} + \frac{R_i^{[0,0,1,1]}}{R_i^{[1,1,0,0]} + R_i^{[0,0,1,1]}} + \frac{R_i^{[1,1,1,0]}}{R_i^{[1,1,0,0]} + R_i^{[1,1,1,0]}} + \\
& \left. \frac{R_i^{[1,0,1,1]}}{R_i^{[1,1,0,0]} + R_i^{[1,0,1,1]}} + \frac{R_i^{[0,1,1,1]}}{R_i^{[1,1,0,0]} + R_i^{[0,1,1,1]}} + \frac{R_i^{[1,1,1,1]}}{R_i^{[1,1,0,0]} + R_i^{[1,1,1,1]}} \right),
\end{aligned} \tag{06}
$$

$$
\begin{aligned}
\mathscr{H}^{[0,0,0,1]}\left(R_i^{[1,0,1,0]}\right) = {} & \frac{\beta S_i R_i^{[1,0,1,0]}}{S_i + R_i^{[1,0,1,0]}} + \frac{\beta S_i R_i^{[1,0,1,1]}}{S_i + R_i^{[1,0,1,1]}} + 2\frac{\beta R^{[1,0,0,0]} R_i^{[0,0,1,0]}}{R^{[1,0,0,0]} + R_i^{[0,0,1,0]}} + \\
& \frac{\beta R^{[1,0,0,0]} R_i^{[1,0,1,0]}}{R^{[1,0,0,0]} + R_i^{[1,0,1,0]}} + \frac{\beta R^{[0,0,1,0]} R_i^{[1,0,1,0]}}{R^{[0,0,1,0]} + R_i^{[1,0,1,0]}} + \frac{\beta R^{[1,0,0,0]} R_i^{[0,0,1,1]}}{R^{[1,0,0,0]} + R_i^{[0,0,1,1]}} + \\
& \frac{\beta R^{[0,0,1,0]} R_i^{[1,0,0,1]}}{R^{[0,0,1,0]} + R_i^{[1,0,0,1]}} + \frac{\beta R^{[1,0,0,0]} R_i^{[1,0,1,1]}}{R^{[1,0,0,0]} + R_i^{[1,0,1,1]}} + \frac{\beta R^{[0,0,1,0]} R_i^{[1,1,0,1]}}{R^{[0,0,1,0]} + R_i^{[1,1,0,1]}} - \\
& \beta R_i^{[1,0,1,0]} \Bigg(\frac{R_i^{[0,1,0,0]}}{R_i^{[0,1,0,0]} + R^{[1,0,1,0]}} + \frac{R_i^{[1,1,0,0]}}{R_i^{[1,1,0,0]} + R_i^{[1,0,1,0]}} + \\
& \frac{R_i^{[0,1,1,0]}}{R_i^{[1,0,1,0]} + R_i^{[0,1,1,0]}} + \frac{R_i^{[0,1,0,1]}}{R_i^{[1,0,1,0]} + R_i^{[0,1,0,1]}} + \frac{R_i^{[1,1,1,0]}}{R_i^{[1,0,1,0]} + R_i^{[1,1,1,0]}} + \\
& \frac{R_i^{[1,1,0,1]}}{R_i^{[1,0,1,0]} + R_i^{[1,1,0,1]}} + \frac{R_i^{[0,1,1,1]}}{R_i^{[1,0,1,0]} + R_i^{[0,1,1,1]}} + \frac{R_i^{[1,1,1,1]}}{R_i^{[1,0,1,0]} + R_i^{[1,1,1,1]}} \Bigg)
\end{aligned} \tag{07}
$$

$$
\begin{aligned}
\mathscr{H}^{[0,0,0,1]}\left(R_i^{[1,0,01]}\right) = {} & \frac{\beta R_i^{[1,0,0,0]} R_i^{[0,0,0,1]}}{R_i^{[1,0,0,0]} + R_i^{[0,0,0,1]}} + \frac{\beta R_i^{[0,0,0,1]} R_i^{[1,0,0,1]}}{R_i^{[0,0,0,1]} + R_i^{[1,0,0,1]}} - \beta R_i^{[1,0,0,1]} \Bigg(\frac{R_i^{[0,1,0,0]}}{R_i^{[1,0,0,1]} + R_i^{[0,1,0,0]}} + \\
& \frac{R_i^{[0,0,1,0]}}{R_i^{[1,0,0,1]} + R_i^{[0,0,1,0]}} + \frac{R_i^{[1,1,0,0]}}{R_i^{[1,0,0,1]} + R_i^{[1,1,0,0]}} + \frac{R_i^{[1,0,1,0]}}{R_i^{[1,0,0,1]} + R_i^{[1,0,1,0]}} + \\
& \frac{R_i^{[0,1,1,0]}}{R_i^{[1,0,0,1]} + R_i^{[0,1,1,0]}} + \frac{R_i^{[0,1,0,1]}}{R_i^{[1,0,0,1]} + R_i^{[0,1,0,1]}} + \frac{R_i^{[0,0,1,1]}}{R_i^{[1,0,0,1]} + R_i^{[0,0,1,1]}} + \\
& \frac{R_i^{[1,1,1,0]}}{R_i^{[1,0,0,1]} + R_i^{[1,1,1,0]}} + \frac{R_i^{[1,1,0,1]}}{R_i^{[1,0,0,1]} + R_i^{[1,1,0,1]}} + \frac{R_i^{[1,0,1,1]}}{R_i^{[1,0,0,1]} + R_i^{[1,0,1,1]}} + \\
& \frac{R_i^{[0,1,1,1]}}{R_i^{[1,0,0,1]} + R_i^{[0,1,1,1]}} + \frac{R_i^{[1,1,1,1]}}{R_i^{[1,0,0,1]} + R_i^{[1,1,1,1]}} \Bigg),
\end{aligned} \tag{08}
$$

$$
\begin{aligned}
\mathscr{H}^{[0,0,0,1]}\left(R_i^{[0,1,1,0]}\right) = {} & \frac{\beta S_i R_i^{[0,1,1,0]}}{S_i + R_i^{[0,1,1,0]}} + \frac{\beta S_i R_i^{[0,1,1,1]}}{S_i + R_i^{[0,1,1,1]}} + 2\frac{\beta R^{[0,1,0,0]} R_i^{[0,0,1,0]}}{R^{[0,1,0,0]} + R_i^{[0,0,1,0]}} + \\
& \frac{\beta R^{[0,1,0,0]} R_i^{[0,1,1,0]}}{R^{[0,1,0,0]} + R_i^{[0,1,1,0]}} + \frac{\beta R^{[0,0,1,0]} R_i^{[0,1,1,0]}}{R^{[0,0,1,0]} + R_i^{[0,1,1,0]}} + \frac{\beta R^{[0,1,0,0]} R_i^{[0,0,1,1]}}{R^{[0,1,0,0]} + R_i^{[0,0,1,1]}} + \\
& \frac{\beta R^{[0,0,1,0]} R_i^{[0,1,0,1]}}{R^{[0,0,1,0]} + R_i^{[0,1,0,1]}} + \frac{\beta R^{[0,1,0,0]} R_i^{[0,1,1,1]}}{R^{[0,1,0,0]} + R_i^{[0,1,1,1]}} + \frac{\beta R^{[0,0,1,0]} R_i^{[0,1,1,1]}}{R^{[0,0,1,0]} + R_i^{[0,1,1,1]}} - \\
& \beta R_i^{[0,1,1,0]} \Bigg(\frac{R_i^{[1,0,0,0]}}{R_i^{[1,0,0,0]} + R^{[0,1,1,0]}} + \frac{R_i^{[1,1,0,0]}}{R_i^{[1,1,0,0]} + R_i^{[0,1,1,0]}} + \\
& \frac{R_i^{[1,0,1,0]}}{R_i^{[1,0,1,0]} + R_i^{[0,1,1,0]}} + \frac{R_i^{[1,0,0,1]}}{R_i^{[1,0,0,1]} + R_i^{[0,1,1,0]}} + \frac{R_i^{[1,1,1,0]}}{R_i^{[0,1,1,0]} + R_i^{[1,1,1,0]}} + \\
& \frac{R_i^{[1,0,1,1]}}{R_i^{[0,1,1,0]} + R_i^{[0,1,1,1]}} + \frac{R_i^{[1,1,0,1]}}{R_i^{[0,1,1,0]} + R_i^{[1,1,0,1]}} + \frac{R_i^{[1,1,1,1]}}{R_i^{[0,1,1,0]} + R_i^{[1,1,1,1]}} \Bigg),
\end{aligned} \tag{09}
$$

$$
\begin{aligned}
\mathscr{H}^{[0,0,0,1]}\left(R_i^{[0,1,0,1]}\right) = {} & \frac{\beta R_i^{[0,1,0,0]} R_i^{[0,0,0,1]}}{R_i^{[0,1,0,0]} + R_i^{[0,0,0,1]}} + \frac{\beta R_i^{[0,0,0,1]} R_i^{[0,1,0,1]}}{R_i^{[0,0,0,1]} + R_i^{[0,1,0,1]}} - \beta R_i^{[0,1,0,1]} \left(\frac{R_i^{[1,0,0,0]}}{R_i^{[1,0,0,0]} + R_i^{[0,1,0,1]}} + \right. \\
& \frac{R_i^{[0,0,1,0]}}{R_i^{[0,0,1,0]} + R_i^{[0,1,0,1]}} + \frac{R_i^{[1,1,0,0]}}{R_i^{[1,1,0,0]} + R_i^{[0,1,0,1]}} + \frac{R_i^{[1,0,1,0]}}{R_i^{[1,0,1,0]} + R_i^{[0,1,0,1]}} + \\
& \frac{R_i^{[1,0,0,1]}}{R^{[1,0,0,1]} + R_i^{[0,1,0,1]}} + \frac{R_i^{[0,1,1,0]}}{R_i^{[0,1,1,0]} R_i^{[0,1,0,1]}} + \frac{R_i^{[0,0,1,1]}}{R_i^{[0,1,0,1]} + R_i^{[0,0,1,1]}} + \\
& \frac{R_i^{[1,1,1,0]}}{R_i^{[0,1,0,1]} + R_i^{[1,1,1,0]}} + \frac{R_i^{[1,1,0,1]}}{R_i^{[0,1,0,1]} + R_i^{[1,1,0,1]}} + \frac{R_i^{[1,0,1,1]}}{R_i^{[0,1,0,1]} + R_i^{[1,0,1,1]}} + \\
& \left. \frac{R_i^{[0,1,1,1]}}{R_i^{[0,1,0,1]} + R_i^{[0,1,1,1]}} + \frac{R_i^{[1,1,1,1]}}{R_i^{[0,1,0,1]} + R_i^{[1,1,1,1]}} \right),
\end{aligned}
\tag{010}
$$

$$
\begin{aligned}
\mathscr{H}^{[0,0,0,1]}\left(R_i^{[0,0,1,1]}\right) = {} & \frac{\beta R_i^{[0,0,1,0]} R_i^{[0,0,0,1]}}{R_i^{[0,0,1,0]} + R_i^{[0,0,0,1]}} + \frac{\beta R_i^{[0,0,0,1]} R_i^{[0,0,1,1]}}{R_i^{[0,0,0,1]} + R_i^{[0,0,1,1]}} - \beta R_i^{[0,0,1,1]} \left(\frac{R_i^{[1,0,0,0]}}{R_i^{[1,0,0,0]} + R_i^{[0,0,1,1]}} + \right. \\
& \frac{R_i^{[0,1,0,0]}}{R_i^{[0,1,0,0]} + R_i^{[0,0,1,1]}} + \frac{R_i^{[1,1,0,0]}}{R_i^{[1,1,0,0]} + R_i^{[0,0,1,1]}} + \frac{R_i^{[1,0,1,0]}}{R_i^{[1,0,1,0]} + R_i^{[0,0,1,1]}} + \\
& \frac{R_i^{[1,0,0,1]}}{R_i^{[1,0,0,1]} + R_i^{[0,0,1,1]}} + \frac{R_i^{[0,1,1,0]}}{R_i^{[0,1,1,0]} + R_i^{[0,0,1,1]}} + \frac{R_i^{[0,1,0,1]}}{R_i^{[0,1,0,1]} + R_i^{[0,0,1,1]}} + \\
& \frac{R_i^{[1,1,1,0]}}{R_i^{[0,0,1,1]} + R_i^{[1,1,1,0]}} + \frac{R_i^{[1,1,0,1]}}{R_i^{[0,0,1,1]} + R_i^{[1,1,0,1]}} + \frac{R_i^{[1,0,1,1]}}{R_i^{[0,0,1,1]} + R_i^{[1,0,1,1]}} + \\
& \left. \frac{R_i^{[0,1,1,1]}}{R_i^{[0,0,1,1]} + R_i^{[0,1,1,1]}} + \frac{R_i^{[1,1,1,1]}}{R_i^{[0,0,1,1]} + R_i^{[1,1,1,1]}} \right),
\end{aligned}
\tag{011}
$$

$$
\begin{aligned}
\mathscr{H}^{[0,0,0,1]}\left(R_i^{[1,1,1,0]}\right) = {} & \beta R_i^{[1,1,1,0]}\left(\frac{S_i}{S_i+R_i^{[1,1,1,0]}} + \frac{R_i^{[1,0,0,0]}}{R_i^{[1,0,0,0]}+R_i^{[1,1,1,0]}} + \frac{R_i^{[0,1,0,0]}}{R_i^{[0,1,0,0]}+R_i^{[1,1,1,0]}} + \right. \\
& \quad \frac{R_i^{[0,0,1,0]}}{R_i^{[0,0,1,0]}+R_i^{[1,1,1,0]}} + \frac{R_i^{[1,1,0,0]}}{R_i^{[1,1,0,0]}+R_i^{[1,1,1,0]}} + \frac{R_i^{[1,0,1,0]}}{R_i^{[1,0,1,0]}+R_i^{[1,1,1,0]}} + \\
& \quad \left.\frac{R_i^{[0,1,1,0]}}{R_i^{[0,1,1,0]}+R_i^{[1,1,1,0]}}\right) + \\
& \beta R_i^{[1,1,0,0]}\left(\frac{R_i^{[0,0,1,0]}}{R_i^{[0,0,1,0]}+R_i^{[1,1,0,0]}} + \frac{R_i^{[1,0,1,0]}}{R_i^{[1,0,1,0]}+R_i^{[1,1,0,0]}} + \frac{R_i^{[0,1,1,0]}}{R_i^{[0,1,1,0]}+R_i^{[1,1,0,0]}}\right) + \\
& \beta R_i^{[1,0,1,0]}\left(\frac{R_i^{[0,1,0,0]}}{R_i^{[0,0,1,0]}+R_i^{[1,0,1,0]}} + \frac{R_i^{[1,1,0,0]}}{R_i^{[1,1,0,0]}+R_i^{[1,0,1,0]}} + \frac{R_i^{[0,1,1,0]}}{R_i^{[0,1,1,0]}+R_i^{[1,0,1,0]}}\right) + \\
& \beta R_i^{[0,1,1,0]}\left(\frac{R_i^{[1,0,0,0]}}{R_i^{[1,0,0,0]}+R_i^{[0,1,1,0]}} + \frac{R_i^{[1,1,0,0]}}{R_i^{[1,1,0,0]}+R_i^{[0,1,1,0]}} + \frac{R_i^{[1,0,1,0]}}{R_i^{[1,0,1,0]}+R_i^{[0,1,1,0]}}\right) + \\
& \frac{\beta R_i^{[1,0,0,0]}R_i^{[0,1,1,0]}}{R_i^{[1,0,0,0]}+R_i^{[0,1,1,0]}} + \frac{\beta R_i^{[0,1,0,0]}R_i^{[1,0,1,0]}}{R_i^{[0,1,0,0]}+R_i^{[1,0,1,0]}} + \frac{\beta R_i^{[0,0,1,0]}R_i^{[1,1,0,0]}}{R_i^{[0,0,1,0]}+R_i^{[1,1,0,0]}} + \\
& \beta R_i^{[1,1,1,1]}\left(\frac{S_i}{S_i+R_i^{[1,1,1,1]}} + \frac{R_i^{[1,0,0,0]}}{R_i^{[1,0,0,0]}+R_i^{[1,1,1,1]}} + \frac{R_i^{[0,1,0,0]}}{R_i^{[0,1,0,0]}+R_i^{[1,1,1,1]}} + \right. \\
& \quad \frac{R_i^{[0,0,1,0]}}{R_i^{[0,0,1,0]}+R_i^{[1,1,1,0]}} + \frac{R_i^{[1,1,0,0]}}{R_i^{[1,1,0,0]}+R_i^{[1,1,1,0]}} + \frac{R_i^{[1,0,1,0]}}{R_i^{[1,0,1,0]}+R_i^{[1,1,1,0]}} + \\
& \quad \left.\frac{R_i^{[0,1,1,0]}}{R_i^{[0,1,1,0]}+R_i^{[1,1,1,0]}}\right) + \\
& \beta R_i^{[1,1,0,1]}\left(\frac{R_i^{[0,0,1,0]}}{R_i^{[0,0,1,0]}+R_i^{[1,1,0,1]}} + \frac{R_i^{[1,0,1,0]}}{R_i^{[1,0,1,0]}+R_i^{[1,1,0,1]}} + \frac{R_i^{[0,1,1,0]}}{R_i^{[0,1,1,0]}+R_i^{[1,1,0,1]}}\right) + \\
& \beta R_i^{[1,0,1,1]}\left(\frac{R_i^{[0,1,0,0]}}{R_i^{[0,0,1,0]}+R_i^{[1,0,1,1]}} + \frac{R_i^{[1,1,0,0]}}{R_i^{[1,1,0,0]}+R_i^{[1,0,1,1]}} + \frac{R_i^{[0,1,1,0]}}{R_i^{[0,1,1,0]}+R_i^{[1,0,1,1]}}\right) + \\
& \beta R_i^{[0,1,1,1]}\left(\frac{R_i^{[1,0,0,0]}}{R_i^{[1,0,0,0]}+R_i^{[0,1,1,1]}} + \frac{R_i^{[1,1,0,0]}}{R_i^{[1,1,0,0]}+R_i^{[0,1,1,1]}} + \frac{R_i^{[1,0,1,0]}}{R_i^{[1,0,1,0]}+R_i^{[0,1,1,1]}}\right) + \\
& \frac{\beta R_i^{[1,0,0,1]}R_i^{[0,1,1,0]}}{R_i^{[1,0,0,1]}+R_i^{[0,1,1,0]}} + \frac{\beta R_i^{[0,1,0,1]}R_i^{[1,0,1,0]}}{R_i^{[0,1,0,1]}+R_i^{[1,0,1,0]}} + \frac{\beta R_i^{[0,0,1,1]}R_i^{[1,1,0,0]}}{R_i^{[0,0,1,1]}+R_i^{[1,1,0,0]}},
\end{aligned}
\tag{012}
$$

$$\begin{aligned}\mathscr{H}^{[0,0,0,1]}\left(R_i^{[1,1,0,1]}\right) = {} & \beta R_i^{[0,0,0,1]}\left(\frac{R_i^{[1,1,0,1]}}{R_i^{[0,0,0,1]}+R_i^{[1,1,0,1]}}+\frac{R_i^{[1,1,0,0]}}{R_i^{[0,0,0,1]}+R_i^{[1,1,0,0]}}\right)+ \\ & \beta R_i^{[1,0,0,1]}\left(\frac{R_i^{[1,1,0,1]}}{R_i^{[1,0,0,1]}+R_i^{[1,1,0,1]}}+\frac{R_i^{[0,1,0,1]}}{R_i^{[1,0,0,1]}+R_i^{[0,1,0,1]}}+\frac{R_i^{[0,1,0,0]}}{R_i^{[1,0,0,1]}+R_i^{[0,1,0,0]}}\right)+ \\ & \beta R_i^{[0,1,0,1]}\left(\frac{R_i^{[1,1,0,1]}}{R_i^{[0,1,0,1]}+R_i^{[1,1,0,1]}}+\frac{R_i^{[1,0,0,1]}}{R_i^{[0,1,0,1]}+R_i^{[1,0,0,1]}}+\frac{R_i^{[1,0,0,0]}}{R_i^{[0,1,0,1]}+R_i^{[1,0,0,0]}}\right)- \\ & \beta R_i^{[1,1,0,1]}\left(\frac{R_i^{[0,0,1,0]}}{R_i^{[0,0,1,0]}+R_i^{[1,1,0,1]}}+\frac{R_i^{[1,0,1,0]}}{R_i^{[1,0,1,0]}+R_i^{[1,1,0,1]}}+\frac{R_i^{[0,1,1,0]}}{R_i^{[0,1,1,0]}+R_i^{[1,1,0,1]}}+\right. \\ & \frac{R_i^{[0,0,1,1]}}{R_i^{[0,0,1,1]}+R_i^{[1,1,0,1]}}+\frac{R_i^{[1,1,1,0]}}{R_i^{[1,1,1,0]}+R_i^{[1,1,0,1]}}+\frac{R_i^{[1,0,1,1]}}{R_i^{[1,0,1,1]}+R_i^{[1,1,0,1]}}+ \\ & \left.\frac{R_i^{[0,1,1,1]}}{R_i^{[0,1,1,1]}+R_i^{[1,1,0,1]}}+\frac{R_i^{[1,1,1,1]}}{R_i^{[1,1,1,1]}+R_i^{[1,1,0,1]}}\right),\end{aligned} \tag{013}$$

$$\begin{aligned}\mathscr{H}^{[0,0,0,1]}\left(R_i^{[1,0,1,1]}\right) = {} & \beta R_i^{[0,0,0,1]}\left(\frac{R_i^{[1,0,1,1]}}{R_i^{[0,0,0,1]}+R_i^{[1,0,1,1]}}+\frac{R_i^{[1,0,1,0]}}{R_i^{[0,0,0,1]}+R_i^{[1,0,1,0]}}\right)+ \\ & \beta R_i^{[1,0,0,1]}\left(\frac{R_i^{[1,0,1,1]}}{R_i^{[1,0,0,1]}+R_i^{[1,0,1,1]}}+\frac{R_i^{[0,0,1,1]}}{R_i^{[1,0,0,1]}+R_i^{[0,0,1,1]}}+\frac{R_i^{[0,0,1,0]}}{R_i^{[1,0,0,1]}+R_i^{[0,0,1,0]}}\right)+ \\ & \beta R_i^{[0,0,1,1]}\left(\frac{R_i^{[1,0,1,1]}}{R_i^{[0,0,1,1]}+R_i^{[1,0,1,1]}}+\frac{R_i^{[1,0,0,1]}}{R_i^{[0,0,1,1]}+R_i^{[1,0,0,1]}}+\frac{R_i^{[1,0,0,0]}}{R_i^{[0,0,1,1]}+R_i^{[1,0,0,0]}}\right)- \\ & \beta R_i^{[1,0,1,1]}\left(\frac{R_i^{[0,1,0,0]}}{R_i^{[0,1,0,0]}+R_i^{[1,0,1,1]}}+\frac{R_i^{[1,1,0,0]}}{R_i^{[1,1,0,0]}+R_i^{[1,0,1,1]}}+\frac{R_i^{[0,1,1,0]}}{R_i^{[0,1,1,0]}+R_i^{[1,0,1,1]}}+\right. \\ & \frac{R_i^{[0,1,0,1]}}{R_i^{[0,1,0,1]}+R_i^{[1,0,1,1]}}+\frac{R_i^{[1,1,1,0]}}{R_i^{[1,1,1,0]}+R_i^{[1,0,1,1]}}+\frac{R_i^{[1,1,0,1]}}{R_i^{[1,1,0,1]}+R_i^{[1,0,1,1]}}+ \\ & \left.\frac{R_i^{[0,1,1,1]}}{R_i^{[0,1,1,1]}+R_i^{[1,0,1,1]}}+\frac{R_i^{[1,1,1,1]}}{R_i^{[1,1,1,1]}+R_i^{[1,0,1,1]}}\right),\end{aligned} \tag{014}$$

$$
\begin{aligned}
\mathscr{H}^{[0,0,0,1]}\left(R_i^{[0,1,1,1]}\right) = \beta R_i^{[0,0,0,1]} &\left(\frac{R_i^{[0,1,1,1]}}{R_i^{[0,0,0,1]}+R_i^{[0,1,1,1]}} + \frac{R_i^{[0,1,1,0]}}{R_i^{[0,0,0,1]}+R_i^{[0,1,1,0]}}\right) + \\
\beta R_i^{[0,1,0,1]} &\left(\frac{R_i^{[0,1,1,1]}}{R_i^{[0,1,0,1]}+R_i^{[0,1,1,1]}} + \frac{R_i^{[0,0,1,1]}}{R_i^{[0,1,0,1]}+R_i^{[0,0,1,1]}} + \frac{R_i^{[0,0,1,0]}}{R_i^{[0,1,0,1]}+R_i^{[0,0,1,0]}}\right) + \\
\beta R_i^{[0,0,1,1]} &\left(\frac{R_i^{[0,1,1,1]}}{R_i^{[0,0,1,1]}+R_i^{[0,1,1,1]}} + \frac{R_i^{[0,1,0,1]}}{R_i^{[0,0,1,1]}+R_i^{[0,1,0,1]}} + \frac{R_i^{[0,1,0,0]}}{R_i^{[0,0,1,1]}+R_i^{[0,1,0,0]}}\right) - \\
\beta R_i^{[0,1,1,1]} &\left(\frac{R_i^{[1,0,0,0]}}{R_i^{[1,0,0,0]}+R_i^{[0,1,1,1]}} + \frac{R_i^{[1,1,0,0]}}{R_i^{[1,1,0,0]}+R_i^{[0,1,1,1]}} + \frac{R_i^{[1,0,1,0]}}{R_i^{[1,0,1,0]}+R_i^{[0,1,1,1]}} + \right. \\
&\frac{R_i^{[1,0,0,1]}}{R_i^{[1,0,0,1]}+R_i^{[0,1,1,1]}} + \frac{R_i^{[1,1,1,0]}}{R_i^{[1,1,1,0]}+R_i^{[0,1,1,1]}} + \frac{R_i^{[1,1,0,1]}}{R_i^{[1,1,0,1]}+R_i^{[0,1,1,1]}} + \\
&\left.\frac{R_i^{[1,0,1,1]}}{R_i^{[1,0,1,1]}+R_i^{[0,1,1,1]}} + \frac{R_i^{[1,1,1,1]}}{R_i^{[1,1,1,1]}+R_i^{[0,1,1,1]}}\right),
\end{aligned}
\tag{015}
$$

$$
\begin{aligned}
&\mathscr{H}^{[0,0,0,1]}\left(R_i^{[1,1,1,1]}\right) = \\
&\quad \beta R_i^{[0,0,0,1]}\Bigg(\frac{R_i^{[1,1,1,0]}}{R_i^{[0,0,0,1]}+R_i^{[1,1,1,0]}}+\frac{R_i^{[1,1,1,1]}}{R_i^{[0,0,0,1]}+R_i^{[1,1,1,1]}}\Bigg)+ \\
&\quad \beta R_i^{[1,0,0,1]}\Bigg(\frac{R_i^{[0,1,1,0]}}{R_i^{[1,0,0,1]}+R_i^{[0,1,1,0]}}+\frac{R_i^{[1,1,1,0]}}{R_i^{[1,0,0,1]}+R_i^{[1,1,1,0]}}+\frac{R_i^{[0,1,1,1]}}{R_i^{[1,0,0,1]}+R_i^{[0,1,1,1]}}+ \\
&\qquad \frac{R_i^{[1,1,1,1]}}{R_i^{[1,0,0,1]}+R_i^{[1,1,1,1]}}\Bigg)+ \\
&\quad \beta R_i^{[0,1,0,1]}\Bigg(\frac{R_i^{[1,0,1,0]}}{R_i^{[0,1,0,1]}+R_i^{[1,0,1,0]}}+\frac{R_i^{[1,1,1,0]}}{R_i^{[1,0,0,1]}+R_i^{[1,1,1,0]}}+\frac{R_i^{[1,0,1,1]}}{R_i^{[1,0,0,1]}+R_i^{[1,0,1,1]}}+ \\
&\qquad \frac{R_i^{[1,1,1,1]}}{R_i^{[0,1,0,1]}+R_i^{[1,1,1,1]}}\Bigg)+ \\
&\quad \beta R_i^{[0,0,1,1]}\Bigg(\frac{R_i^{[1,1,0,0]}}{R_i^{[0,0,1,1]}+R_i^{[1,1,00]}}+\frac{R_i^{[1,1,1,0]}}{R_i^{[0,0,1,1]}+R_i^{[1,1,1,0]}}+\frac{R_i^{[1,1,0,1]}}{R_i^{[0,0,0,1]}+R_i^{[1,1,0,1]}}+ \\
&\qquad \frac{R_i^{[1,1,1,1]}}{R_i^{[0,0,1,1]}+R_i^{[1,1,1,1]}}\Bigg)+ \\
&\quad \beta R_i^{[1,1,0,1]}\Bigg(\frac{R_i^{[0,0,1,0]}}{R_i^{[0,0,1,0]}+R_i^{[1,1,0,1]}}+\frac{R_i^{[1,0,1,0]}}{R_i^{[1,0,1,0]}+R_i^{[1,1,0,1]}}+\frac{R_i^{[0,1,1,0]}}{R_i^{[0,1,1,0]}+R_i^{[1,1,0,1]}}+ \\
&\qquad \frac{R_i^{[0,0,1,1]}}{R_i^{[0,0,1,1]}+R_i^{[1,1,0,1]}}+\frac{R_i^{[1,1,1,0]}}{R_i^{[1,1,1,0]}+R_i^{[1,1,0,1]}}+\frac{R_i^{[1,0,1,1]}}{R_i^{[1,0,1,1]}+R_i^{[1,1,0,1]}}+ \\
&\qquad \frac{R_i^{[0,1,1,1]}}{R_i^{[1,1,0,1]}+R_i^{[0,1,1,1]}}+\frac{R_i^{[1,1,1,1]}}{R_i^{[1,1,1,1]}+R_i^{[1,1,0,1]}}\Bigg)+ \\
&\quad \beta R_i^{[1,0,1,1]}\Bigg(\frac{R_i^{[0,1,0,0]}}{R_i^{[0,1,0,0]}+R_i^{[1,0,1,1]}}+\frac{R_i^{[1,1,0,0]}}{R_i^{[1,1,0,0]}+R_i^{[1,0,1,1]}}+\frac{R_i^{[0,1,1,0]}}{R_i^{[0,1,1,0]}+R_i^{[1,0,1,1]}}+ \\
&\qquad \frac{R_i^{[0,1,0,1]}}{R_i^{[0,1,0,1]}+R_i^{[1,0,1,1]}}+\frac{R_i^{[1,1,1,0]}}{R_i^{[1,1,1,0]}+R_i^{[1,0,1,1]}}+\frac{R_i^{[1,1,0,1]}}{R_i^{[1,1,0,1]}+R_i^{[1,0,1,1]}}+ \\
&\qquad \frac{R_i^{[0,1,1,1]}}{R_i^{[1,0,1,1]}+R_i^{[0,1,1,1]}}+\frac{R_i^{[1,1,1,1]}}{R_i^{[1,1,1,1]}+R_i^{[1,0,1,1]}}\Bigg)+ \\
&\quad \beta R_i^{[0,1,1,1]}\Bigg(\frac{R_i^{[1,0,0,0]}}{R_i^{[1,0,0,0]}+R_i^{[0,1,1,1]}}+\frac{R_i^{[1,1,0,0]}}{R_i^{[1,1,0,0]}+R_i^{[0,1,1,1]}}+\frac{R_i^{[1,0,1,0]}}{R_i^{[1,0,1,0]}+R_i^{[0,1,1,1]}}+ \\
&\qquad \frac{R_i^{[1,0,0,1]}}{R_i^{[1,0,0,1]}+R_i^{[0,1,1,1]}}+\frac{R_i^{[1,1,1,0]}}{R_i^{[1,1,1,0]}+R_i^{[0,1,1,1]}}+\frac{R_i^{[1,1,0,1]}}{R_i^{[1,1,0,1]}+R_i^{[0,1,1,1]}}+ \\
&\qquad \frac{R_i^{[1,0,1,1]}}{R_i^{[1,0,1,1]}+R_i^{[0,1,1,1]}}+\frac{R_i^{[1,1,1,1]}}{R_i^{[1,1,1,1]}+R_i^{[0,1,1,1]}}\Bigg).
\end{aligned}
\tag{016}
$$

Therefore the farm flow model when cefelexin resistance is chromosomally encoded is defined by the system of equations given by (01)-(021), (01)-(017).

$$\frac{dR_i^{[x_1,x_2,x_3,x_4]}}{dt} = \mathscr{F}(R_i^{[x_1,x_2,x_3,x_4]}) + \mathscr{D}(R_i^{[x_1,x_2,x_3,x_4]}) + \mathscr{G}(R_i^{[x_1,x_2,x_3,x_4]}) + \mathscr{H}^{[0,0,0,1]}(R_i^{[x_1,x_2,x_3,x_4]}),$$
$$\text{where } i \in \{\text{dairy, heifer, UR, muck, eff., tank}\} \ \& \ [x_1,x_2,x_3,x_4] \in \{0,1\}^4. \quad (017)$$

where $\mathscr{F}(R_i^{[x_1,x_2,x_3,x_4]})$, $\mathscr{D}(R_i^{[x_1,x_2,x_3,x_4]})$, $\mathscr{G}(R_i^{[x_1,x_2,x_3,x_4]})$ are defined by (019), (020) and (021) respectively.

Model Parameters

Supplementary Table 1: Volume Flow Parameters

Parameter	Parameter Name	Parameter Values	Units	Source
a	Main dairy shed waste volume input	1.238×10^3	L h^{-1}	farm observations, [1]
b	Bulling heifer shed waste volume input	1.358×10^2	L h^{-1}	farm observations, [1]
ρ	Scraper channel natural outflow rate	4.167×10^{-2}	h^{-1}	Assumed
γ	Pump rate from UR to slurry tank	9.625×10^{-2}	h^{-1}	farm observations, [2]
σ	Pump rate from UR to scraper channels	4.010×10^{-3}	h^{-1}	farm observations, [2]
ε	Fraction of slurry separated as liquid	0.950	-	farm observations
κ_{muck}	Muck heap emptying rate	7.500×10^{-5}	h^{-1}	farm observations
η	Muck heap effluent run off rate	2.083×10^{-5}	h^{-1}	farm observations
ι_{silage}	Volume of effluent run off from the silage clamp	2.382	L h^{-1}	farm observations

Supplementary Table 2: Metal Parameters

Parameter	Parameter Name	Parameter Values	Units	Source
$a_{\text{feed}}^{[Cu]}$	Copper input from daily cow feed in main dairy shed	2.985×10^3	mg h^{-1}	farm observations, [3, 4]
$a_{\text{feed}}^{[Zn]}$	Zinc input from daily cow feed in main dairy shed	1.090×10^4	mg h^{-1}	farm observations, [5, 4]
$b_{\text{feed}}^{[Cu]}$	Copper input from daily cow feed in bulling heifer shed	8.954×10^2	mg h^{-1}	farm observations, [3, 4]
$b_{\text{feed}}^{[Zn]}$	Zinc input from daily cow feed in bulling heifer shed	3.269×10^3	mg h^{-1}	farm observations, [5, 4]

Supplementary Table 3: Antibiotic Parameters

Parameter	Parameter Name	Parameter Values	Units	Source
$\delta^{[Oxy]}$	Oxytetracycline degradation rate	3.269×10^3	h^{-1}	[6]
$\delta^{[Cex]}$	Cefalexin degradation rate	3.269×10^3	h^{-1}	[6]

Supplementary Table 4: Bacterial Parameters

Parameter	Parameter Name	Parameter Values	Units	Source
r	Specific growth rate	8.000 $\times 10^{-2}$	h^{-1}	[6]
β	Horizontal gene transfer rate	1.000 $\times 10^{-6}$	h^{-1}	[6]
N_{Max}	Carrying capacity	1.000 $\times 10^{10}$	CFU L^{-1}	[6]
δ	Natural death rate of bacteria	4.684 $\times 10^{-2}$	h^{-1}	Estimated, [6]
$\psi_{E.coli}$	Concentration of bacteria in slurry inflow	4.479 $\times 10^{7}$	CFU L^{-1} h^{-1}	[6]
ν	Proportion of resistant bacteria in slurry inflow	3.178 $\times 10^{-4}$	-	Estimated, [6]
$\alpha^{[Cu]}$	Fitness cost of copper resistance carried on plasmid	2.921 $\times 10^{-1}$	-	Estimated, [6]
$\alpha^{[Zn]}$	Fitness cost of zinc resistance carried on plasmid	2.921 $\times 10^{-1}$	-	Estimated, [6]
$\alpha^{[Oxy]}$	Fitness cost of Oxytetracycline resistance carried on plasmid	3.000 $\times 10^{-3}$	-	[6]
$\alpha^{[Cex]}$	Fitness cost of Cefalexin resistance carried on plasmid	1.561 $\times 10^{-1}$	-	Estimated, [6]

Supplementary Table 5: Pharmacodynamic Parameters

Parameter	Parameter Name	Parameter Values	Units	Source
$MIC^{[Cu]}$	Minimum inhibitory concentration of copper	212.79	mg L^{-1}	[7, 8, 6]
$MIC^{[Zn]}$	Minimum inhibitory concentration of zinc	2760.31	mg L^{-1}	[7, 8, 6]
$MIC^{[Oxy]}$	Minimum inhibitory concentration of Oxytetracycline	1	mg L^{-1}	[6]
$MIC^{[Cex]}$	Minimum inhibitory concentration of Cefalexin	8	mg L^{-1}	[6]
$E_{Max}^{[Cu]}$	Maximum death rate due to copper	1.74	h^{-1}	[7, 8, 6]
$E_{Max}^{[Zn]}$	Maximum death rate due to zinc	1.37	h^{-1}	[7, 8, 6]
$E_{Max}^{[Oxy]}$	Maximum death rate due to Oxytetracycline	1	h^{-1}	[6]
$E_{Max}^{[Cex]}$	Maximum death rate due to Cefalexin	1	h^{-1}	[6]
$H^{[Cu]}$	Hill coefficient for copper	1.54	-	[7, 8, 6]
$H^{[Zn]}$	Hill coefficient for zinc	0.72	-	[7, 8, 6]
$H^{[Oxy]}$	Hill coefficient for Oxytetracycline	2	-	[6]
$H^{[Cex]}$	Hill coefficient for Cefalexin	2	-	[6]

Supplementary Table 6: Discrete Parameters

Parameter	Parameter Name	Parameter Values	Units	Source
T_{footbath}	Days on which metal footbaths are emptied into the main dairy shed scraper channels	$\{7, 14, 21, \ldots, 364\}$	days	Assumed
$T_{\text{extra foot.}}$	Days on which additional metal footbaths are emptied into the main dairy shed scraper channels	$\{21, 42, 63, \ldots, 357\}$	days	Assumed
$T_{\text{eff. flushing}}$	Days on which MHE is used to flush out the main dairy shed and bulling heifer shed scraper channels	$\{28, 56, 74, \ldots, 364\}$	days	Assumed
$T_{\text{Empty Tank}}$	Days on which the slurry tank is emptied	$\{50, 110, 170, \ldots, 350\}$	days	Assumed, [6]

Reasonable Parameter Ranges for Sensitivity Analyses

Supplementary Table 7: continuous action parameters

Parameter	Parameter Name	Parameter Value	Reasonable Parameter Space
$\alpha^{[metal]}$	Fitness cost of metal resistance	2.921×10^{-1}	$[0,1]$
$\alpha^{[Oxy]}$	Fitness cost of oxytetracycline resistance	3.000×10^{-3}	$[0,1]$
$\delta^{[Oxy]}$	Degradation rate of oxytetracycline	2.888×10^{-3}	$\left[1 \times 10^{-5}, 1 \times 10^{-1}\right]$
$\alpha^{[Cex]}$	Fitness cost of cefelexin resistance	1.561×10^{-1}	$[0,1]$
$\delta^{[Cex]}$	Degradation rate of cefelexin	1.764×10^{-3}	$\left[1 \times 10^{-5}, 1 \times 10^{-1}\right]$
ν	Proportion of resistant bacteria in waste inflow	3.178×10^{-4}	$[0, 0.3]$
r	Bacterial growth rate	8.000×10^{-2}	$[0, 0.9]$
β	Horizontal gene transfer rate	1.000×10^{-6}	$\left[1 \times 10^{-9}, 1 \times 10^{-2}\right]$
δ	Bacterial environmental death rate	4.684×10^{-2}	$\left[1.250 \times 10^{-2}, 3.360 \times 10^{-1}\right]$

Table 07: This table shows the farm flow model bacterial and antimicrobial parameters that we have explored in our parameter sensitivity analysis, Fig 3.3, and gives the parameter space which the parameters were sampled from in this analysis.

Supplementary Table 8: discrete action parameters

Parameter	Parameter Name	Parameter Value	Reasonable Parameter Space
τ_{tank}	Frequency of emptying of the slurry tank	60	$[0, 365]$
τ_{footbath}	Frequency of emptying of the main dairy shed metal footbaths	7	$[0, 100]$
$V^{[footbath]}$	Volume of main dairy shed metal footbaths	800	$[50, 5000]$
$\tau_{\text{eff. flushing}}$	Frequency of muck heap effluent flushing of the main dairy shed and bulling heifer shed scraper channels	28	$[0, 365]$
ω	Volume of muck heap effluent used in flushing of scraper channels	1.182×10^{4}	$\left[1 \times 10^{2}, 1 \times 10^{5}\right]$

Table 08: This table shows the farm flow model discrete farm management parameters that we have explored in our parameter sensitivity analysis, Fig 3.5, and gives the parameter space from which the parameters were sampled.

Supplementary Table 9: Genomic *ampC* analysis: WT strains

Strain	Location	Accession	Resistance Phenotyopes	*ampC* Mutations	Variant
49	ST	GCA_030501425.1	AMC, AMP, C, CPD, (CTX), (S10), SXT, TE	NONE	0
55	ST	GCA_022489665.1	AMC, AMP, C,CPD, (CTX), (S10), SXT	NONE	0
308	ST	GCA_030501235.1	AMP, CIP, CPD, CTX, F, FOX, NA, S10, SXT	NONE	0
687	ST	GCA_030500955.1	CTX, CAZ, AZM, TE, ATM, AMP, (S10), CPD, (CIP)	NONE	0
726	ST	GCA_030500995.1	AMP, ATM, CAZ, CIP, CPD, CTX, (NA), (S10), TE	NONE	0
869	UR	GCA_030501395.1	AMC, AMP, (CAZ), CPD, CTX, FOX	NONE	0
875	GHS	GCA_030500975.1	AMP, ATM, AZM, CAZ, (CIP), CPD, CTX, (NA), (S10), TE	NONE	0
876	GHS	GCA_030500965.1	AMP, AZM, CAZ, (CIP), CPD, CTX, FOX	NONE	0
939	MHE	GCA_030501035.1	AMP, ATM, CAZ, (CIP), CPD, CTX, (S10), TE	NONE	0
947	MHE	GCA_030501055.1	AMP, ATM, CAZ, (CIP), CPD, CTX, (NA), (S10), TE	NONE	0
956	MHE	GCA_030501065.1	AMP, ATM, AZM, CAZ, (CIP), CPD, CTX, (NA), (S10)	NONE	0
961	DSSC	GCA_030501085.1	AMP, ATM, AZM, CAZ, (CIP), CPD, CTX, (IPM), (S10), TE	NONE	0
962	DSSC	GCA_030501075.1	AMP, ATM, AZM, CAZ, (CIP), CPD, CTX, (S10), SXT, TE	NONE	0
965	DSSC	GCA_030501135.1	AMP, ATM, CAZ, (CIP), CPD, CTX, TE	NONE	0

Table 09: Details of the 14 wild type *ampC* strains of 31 whole genome sequenced strains from Baker *et al.* 2022, alongside measured resistance phenotypes. All 14 of these strains are identified as ESCR. Locations are: DLO = Dairy Lane Outside; DSSC = Dairy Shed Scraper Channel; GHS = Growing Heifer Shed; MHE = Muck Heap Effluent; ST = Slurry Tank; UR = Underground Reservoir; WHSSC = Weaned Heifer Shed Scraper Channel. Phenotypic resistances are: AMC = Amoxicillin-Clavulanic Acid; AMP = Ampicillin; ATM = Aztreonam; AZM = Azithromycin; C = Chloramphenicol; CAZ = Ceftazidime; CIP = Ciprofloxacin; CPD = Cefpodoxime; CTX = Cefotazime; F = Nitrofurantoin; FOX = Cefotaxime; IPM = Imipenem; NA = Nalidixic Acid; S10 = Streptomycin; SXT = Trimethoprim-Sulfamethoxazole; TE = Oxytetracycline. Phenotypes in parentheses () indicate intermediate results.

Supplementary Table 10: Genomic *ampC* analysis: mutant strains

Strain	Location	Accession	Resistance Phenotyopes	*ampC* Mutations	Variant
56	ST	GCA_022489605.1	AMC, AMP, C, CPD, (CTX), (S10), SXT	70(T)	1
67	ST	GCA_022489505.1	AMP, S10	70(T)	1
397	ST	GCA_022489555.1	AMP, CAZ, CIP, S10	70(T)	1
774	ST	GCA_030501415.1	CTX, CAZ, TE, ATM, AMP, CPD, (CIP)	70(T)	1
51	ST	GCA_022489585.1	AMC, AMP, C, (CPD), CTX, (S10), SXT	-18(A) -1(T) 58(T)	2
99	ST	GCA_030501155.1	AMP, C, CPD, CTX, IPM, S10, TE	-18(A) -1(T) 58(T)	2
113	ST	GCA_022489535.1	AMC, AZM, (CAZ), CIP, (S10), TE	-18(A) -1(T) 58(T)	2
183	ST	GCA_030501175.1	CIP, CPD, CTX, F, FOX, S10	-18(A) -1(T) 58(T)	2
295	ST	GCA_030501205.1	AMC, AMP, ATM, (CIP), CTX, FOX, NA, S10	-18(A) -1(T) 58(T)	2
408	ST	GCA_030501255.1	CPD, CTX, FOX, (S10), SXT	-18(A) -1(T) 58(T)	2
518	ST	GCA_030501295.1	AMC, AMP, (ATM), C, CAZ, CPD, CTX, FOX, NA	-18(A) -1(T) 58(T)	2
582	ST	GCA_030501305.1	AMC, AMP, AZM, CAZ, CIP, (CPD), (CTX), F, FOX, NA, S10, SXT	-18(A) -1(T) 58(T)	2
486	ST	GCA_030501265.1	AMC, AMP, C, CIP, CPD, (CTX), FOX, S10, SXT, TE	-42(T) -18(A) -1(T) 58(T)	3
825	DLO	GCA_030501315.1	CIP, CPD, CTX, FOX	-42(T) -18(A) -1(T) 58(T)	3
826	GHS	GCA_030501355.1	AMC, AMP, C, CAZ, CPD, CTX, FOX, SXT	-42(T) -18(A) -1(T) 58(T)	3
867	BHSSC	GCA_030501365.1	AMC, AMP, (ATM), AZM, CAZ, CIP, CPD, CTX, FOX, (S10), SXT	-42(T) -18(A) -1(T) 58(T)	3
127	ST	GCA_030501165.1	AZM, CAZ, CPD, (CTX), F, FOX, NA, S10, SXT	22(T) 26(G) 27(T) 32(A)	4

Table 010: Details of the 17 chromosomal *ampC* mutations of 31 whole genome sequenced strains from Baker *et al.* 2022 [6], alongside measured resistance phenotypes. 16 of these 17 strains are identified as ESCR, the exception being strain 67. There are four variants of *ampC* mutations; strain 67 is also Variant 1. Locations are: DLO = Dairy Lane Outside; DSSC = Dairy Shed Scraper Channel; GHS = Growing Heifer Shed; MHE = Muck Heap Effluent; ST = Slurry Tank; UR = Underground Reservoir; BHSSC = Bulling Heifer Shed Scraper Channel. Phenotypic resistances are: AMC = Amoxicillin-Clavulanic Acid; AMP = Ampicillin; ATM = Aztreonam; AZM = Azithromycin; C = Chloramphenicol; CAZ = Ceftazidime; CIP = Ciprofloxacin; CPD = Cefpodoxime; CTX = Cefotazime; F = Nitrofurantoin; FOX = Cefotaxime; IPM = Imipenem; NA = Nalidixic Acid; S10 = Streptomycin; SXT = Trimethoprim-Sulfamethoxazole; TE = Oxytetracycline. Phenotypes in parentheses () indicate intermediate results. The wild type DNA bases at mutated positions are: -42(C); -18(G); -1(C); 22(C); 26(T); 27(A); 32(G); 58(C); 70(C).

Supplementary Figures

Supplementary Figure 1: model scheme for chromosomal carriage of cefalexin resistance genes

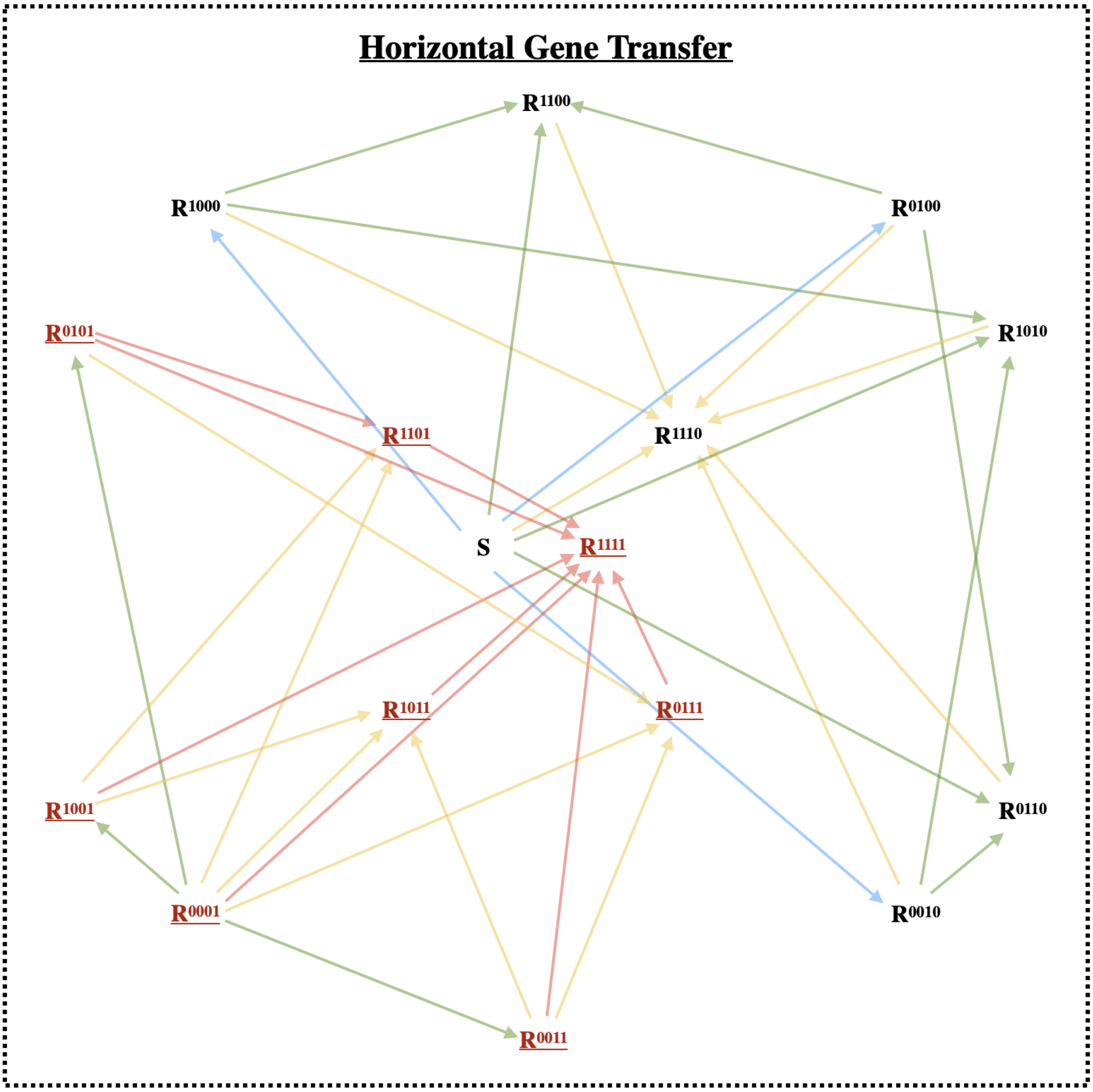

Figure 01: Schematic diagram describing the horizontal gene transfer dynamics in the case where cefalexin resistance is encoded chromosomally (rather than on a plasmid). As cex-resistance is located on the chromosome, bacteria carrying cex-resistance do not incur a fitness cost . Metal and oxytetracycline resistances are still modelled as plasmid mediated and transfer of these resistances can occur horizontally. However, cex-resistance cannot be transferred horizontally. Bacteria labelled in red denote populations which have cefalexin-resistance, while bacteria labelled in black do not.

References

[1] American Society of Agricultural Engineers. Manure Production and Characteristics. Technical report, American Society of Agricultural Engineers, 2005.

[2] Landia. *Submersible Slurry Pump DG*, n.d.

[3] EFSA Panel on Additives and Products or Substances used in Animal Feed (FEEDAP) . Scientific opinion on the safety and efficacy of copper compounds (e4) as feed additives for all animal species (cupric acetate, monohydrate; basic cupric carbonate, monohydrate; cupric chloride, dihydrate; cupric oxide; cupric sulphate, pentahydrate; cupric chelate of amino acids, hydrate; cupric chelate of glycine, hydrate), based on a dossier submitted by fefana asbl. *EFSA Journal*, 13(4), 2015.

[4] INRA-CIRAD-AFZ. Feedtables: Tables of composition and nutritional values of feed materials. https://feedtables.com/, 2018. [Online; accessed 31-May-2019].

[5] EFSA Panel on Additives and Products or Substances used in Animal Feed (FEEDAP) . Scientific opinion on the safety and efficacy of zinc compounds (e6) as feed additives for all animal species (zinc acetate, dihydrate; zinc chloride, anhydrous; zinc oxide; zinc sulphate, heptahydrate; zinc sulphate, monohydrate; zinc chelate of amino acids, hydrate; zinc chelate of glycine, hydrate), based on a dossier submitted by fefana asbl. *EFSA Journal*, 13(4), 2015.

[6] Michelle Baker, Alexander D. Williams, Steven P.T. Hooton, Richard Helliwell, Elizabeth King, Thomas Dodsworth, Rosa María Baena-Nogueras, Andrew Warry, Catherine A. Ortori, Henry Todman, Charlotte J. Gray-Hammerton, Alexander C.W. Pritchard, Ethan Iles, Ryan Cook, Richard D. Emes, Michael A. Jones, Theodore Kypraios, Helen West, David A. Barrett, Stephen J. Ramsden, Rachel L. Gomes, Chris Hudson, Andrew D. Millard, Sujatha Raman, Carol Morris, Christine E.R. Dodd, Jan-Ulrich Kreft, Jon L. Hobman, and Dov J. Stekel. Antimicrobial resistance in dairy slurry tanks: A critical point for measurement and control. *Environment International*, 169:107516, 2022.

[7] A Ivask, T Rolova, and Kahru A. A suite of recombinant luminescent bacterial strains for the quantification of bioavailable heavy metals and toxicity testing. *BMC Biotechnology*, 9(1), 2009.

[8] Sankalp Arya, Alexander Williams, Saul Vazquez Reina, Charles W. Knapp, Jan-Ulrich Kreft, Jon L. Hobman, and Dov J. Stekel. Towards a general model for predicting minimal metal concentrations co-selecting for antibiotic resistance plasmids. *Environmental Pollution*, 275, 2021.
