# Supplementary material for: Modelling the impact of wastewater flows and management practices on antimicrobial resistance in dairy farms
Source: NPJ Antimicrob Resist. 2024 May 14;2:13. doi: 10.1038/s44259-024-00029-4 (PMC11093733; doi:10.1038/s44259-024-00029-4)
Supplement: Supplementary file 5 — Reporting Summary [file 44259_2024_29_MOESM5_ESM.pdf]

nature portfolio

Corresponding author(s): Dov Stekel

Last updated by author(s): Jan 15, 2024

Reporting Summary

Nature Portfolio wishes to improve the reproducibility of the work that we publish. This form provides structure for consistency and transparency in reporting. For further information on Nature Portfolio policies, see our Editorial Policies and the Editorial Policy Checklist.

Statistics

For all statistical analyses, confirm that the following items are present in the figure legend, table legend, main text, or Methods section.

n/a	Confirmed	
☒	☐	The exact sample size (*n*) for each experimental group/condition, given as a discrete number and unit of measurement
☒	☐	A statement on whether measurements were taken from distinct samples or whether the same sample was measured repeatedly
☒	☐	The statistical test(s) used AND whether they are one- or two-sided<br>*Only common tests should be described solely by name; describe more complex techniques in the Methods section.*
☒	☐	A description of all covariates tested
☒	☐	A description of any assumptions or corrections, such as tests of normality and adjustment for multiple comparisons
☒	☐	A full description of the statistical parameters including central tendency (e.g. means) or other basic estimates (e.g. regression coefficient) AND variation (e.g. standard deviation) or associated estimates of uncertainty (e.g. confidence intervals)
☒	☐	For null hypothesis testing, the test statistic (e.g. *F*, *t*, *r*) with confidence intervals, effect sizes, degrees of freedom and *P* value noted<br>*Give P values as exact values whenever suitable.*
☒	☐	For Bayesian analysis, information on the choice of priors and Markov chain Monte Carlo settings
☒	☐	For hierarchical and complex designs, identification of the appropriate level for tests and full reporting of outcomes
☒	☐	Estimates of effect sizes (e.g. Cohen's *d*, Pearson's *r*), indicating how they were calculated

Our web collection on statistics for biologists contains articles on many of the points above.

Software and code

Policy information about availability of computer code

Data collection	NA
Data analysis	Simulations carried out in Matlab version R2020. The Matlab code for the model with plasmid and chromosomal gene carriage are supplied as Supplementary Code 1 and 2 respectively.

For manuscripts utilizing custom algorithms or software that are central to the research but not yet described in published literature, software must be made available to editors and reviewers. We strongly encourage code deposition in a community repository (e.g. GitHub). See the Nature Portfolio guidelines for submitting code & software for further information.

Data

Policy information about availability of data

All manuscripts must include a data availability statement. This statement should provide the following information, where applicable:

- Accession codes, unique identifiers, or web links for publicly available datasets
- A description of any restrictions on data availability
- For clinical datasets or third party data, please ensure that the statement adheres to our policy

The microbiology count data used for Figure 5 is supplied as Supplementary Data File 1

Research involving human participants, their data, or biological material

Policy information about studies with human participants or human data. See also policy information about sex, gender (identity/presentation), and sexual orientation and race, ethnicity and racism.

Reporting on sex and gender	NA
Reporting on race, ethnicity, or other socially relevant groupings	NA
Population characteristics	NA
Recruitment	NA
Ethics oversight	NA

Note that full information on the approval of the study protocol must also be provided in the manuscript.

Field-specific reporting

Please select the one below that is the best fit for your research. If you are not sure, read the appropriate sections before making your selection.

☐ Life sciences ☐ Behavioural & social sciences ☒ Ecological, evolutionary & environmental sciences

For a reference copy of the document with all sections, see nature.com/documents/nr-reporting-summary-flat.pdf

Ecological, evolutionary & environmental sciences study design

All studies must disclose on these points even when the disclosure is negative.

Study description	The main focus of this study is a mathematical model / simulation to explore the impact of wastewater flows and management practices on antimicrobial resistance (AMR) in slurry. A small amount of additional microbiological data on Escherichia coli counts from different parts of the dairy farm is included as part of the findings.
Research sample	Liquid samples were collected from different areas on the farm on two different dates. On 21st November 2017, samples were taken from the main dairy cubicle shed (dairy lane inside, dairy lane outside the scraper channel), bulling heifer cubicle shed and scraper channel, underground reservoir, the muck heap effluent tank and the slurry tank. On 12th December 2017, samples were taken from the bulling heifer shed (as before), the sheds containing weaned and growing heifers (not in Figure 1), straw from the weaned heifer shed, muck heap straw, the muck heap effluent tank and the slurry tank. E. coli strains were isolated using Tryptone Bile X-Glucuronide (TBX) or MacConkey agar or TBX/MacConkey supplemented with 16 g ml-1 ampicillin (AMP), or 2 g ml-1 cefotaxime (CTX); or on CHROMagar ESBLTM agar, as described previously22. The CTX and CHROMagar plates were specifically used because ESBL-EC are the WHO recommended indicator strains25. Putative E. coli isolates were subcultured onto TBX agar or TBX agar supplemented with 2 g ml-1 CTX. E. coli strains were confirmed using oxidase and catalase tests as described23.
Sampling strategy	The rationale was to try to find ESCR E. coli in different farm locations. This was in support of a much larger scale collection and characterisation of Escherichia coli from the slurry tank of the farm on 17 different dates that was previously reported in Ref. 23.
Data collection	As above. Data collection and characterization were carried out by co-author Liz King under supervision of co-author Jon Hobman
Timing and spatial scale	Dates as above
Data exclusions	None
Reproducibility	All samples were taken in triplicate. All data points are shown on Figure 5.
Randomization	NA
Blinding	NA

Did the study involve field work? ☐ Yes ☒ No

Reporting for specific materials, systems and methods

We require information from authors about some types of materials, experimental systems and methods used in many studies. Here, indicate whether each material, system or method listed is relevant to your study. If you are not sure if a list item applies to your research, read the appropriate section before selecting a response.

Materials & experimental systems

n/a	Involved in the study
☒	☐ Antibodies
☒	☐ Eukaryotic cell lines
☒	☐ Palaeontology and archaeology
☒	☐ Animals and other organisms
☒	☐ Clinical data
☒	☐ Dual use research of concern
☒	☐ Plants

Methods

n/a	Involved in the study
☒	☐ ChIP-seq
☒	☐ Flow cytometry
☒	☐ MRI-based neuroimaging

Plants

Seed stocks	NA
Novel plant genotypes	NA
Authentication	NA
